# Combining Asymmetric PCR with PAM-Independent Cas12a Analysis of Single-Stranded Amplicons for On-Site Nucleic Acid Testing

**DOI:** 10.3390/bios16090512

**Published:** 2026-09-11

**Authors:** Konstantin G. Ptitsyn, Olga S. Timoshenko, Svetlana A. Khmeleva, Leonid K. Kurbatov, Adelina A. Stepanova, Evgenia M. Nikiforova, Elena V. Suprun, Sergey P. Radko, Andrey V. Lisitsa

**Affiliations:** 1 V.N. Orekhovich Institute of Biomedical Chemistry, 10 Pogodinskaya St., 119121 Moscow, Russia; konstantin157@yandex.ru (K.G.P.); ryzhakova.olga@list.ru (O.S.T.); diny1204@yandex.ru (S.A.K.); leonid15@mail.ru (L.K.K.); adelina.a.stepanova@yandex.ru (A.A.S.); nikiforova-em@yandex.ru (E.M.N.); lisitsa052@gmail.com (A.V.L.); 2 The Chemistry Faculty, M.V. Lomonosov Moscow State University, 1/3 Lenin Hills, 119991 Moscow, Russia; lenasuprun@mail.ru

**Keywords:** on-site nucleic acid testing, asymmetric PCR, single-stranded DNA, Cas12a nuclease, protospacer adjacent motif, uracil-containing amplicons, visual readout, *P. polaris* detection

## Abstract

A biosensing system relying on asymmetric PCR (aPCR) and PAM-independent recognition of single-stranded DNA amplicons by a Cas12a/gRNA complex is adapted for on-site nucleic acid testing (NAT). aPCR was performed on a low-cost, portable, smartphone-controlled thermocycler operating in endpoint mode. Subsequently, a selective visual readout of the aPCR outcome was achieved using a Cas12a-based assay. A mechanism to prevent false positives was incorporated into the aPCR/Cas12a biosensing system by introducing uracil into the amplicons produced during amplification. *Pectobacterium* species—harmful bacterial plant pathogens—and genus-specific primers Y1 and Y2 against *Pectobacterium* spp. were utilized as a convenient model. The absence of a PAM requirement allowed for fine-tuning the selectivity of the Cas12a assay against a strain of a particular *Pectobacterium* species, *P. polaris*, by properly positioning the gRNA spacer on the ssDNA. *P. polaris* was detected down to 10 copies of the bacterial genome per reaction, with an overall testing time of about 2 h and either instrumental or visual readouts. Similar selectivity and sensitivity toward a *P. polaris* strain were observed with the aPCR/Cas12a biosensing system for potato samples artificially contaminated with *Pectobacterium* species. The overall findings indicate aPCR/Cas12a-based biosensing as a technique suitable for on-site NAT.

## 1. Introduction

Over the last several years, the combination of CRISPR/Cas nucleases with methods of nucleic acid amplification has appeared as an emerging trend in the development of programmable biosensing systems for on-site nucleic acid testing (NAT). Among CRISPR nucleases, Cas12 nucleases were most often used and commonly coupled to isothermal amplifications such as recombinant polymerase amplification (RPA, Cas12a nuclease) and loop-mediated isothermal amplification (LAMP, Cas12a or Cas12b nucleases) [1,2,3]. Alongside the methods of isothermal amplification, Cas12a nuclease was also employed in conjunction with end-point polymerase chain reaction (PCR) for selective post-PCR analysis of target amplicons [3,4]. The Cas12a-based readout of PCR outcomes is technically much simpler than electrophoretic analysis of PCR products and offers additional selectivity compared to the use of lateral flow strips. Initially, PCR/Cas12a detection was considered an advanced mode of endpoint PCR for laboratory settings [3]. However, the emerging availability of low-cost, portable thermocyclers, which rely on end-point detection and operate via a smartphone application [5], provided the ground for converting PCR/Cas12a biosensing into a technique also suitable for on-site NAT.

The bottleneck in the design of amplification-based Cas12a biosensing systems is a requirement for a protospacer adjacent motif (PAM) in double-stranded amplicons. This imposes an additional restriction on primers so that the most effective primer pairs cannot necessarily be utilized. Moreover, the PAM requirement limits the choice of spacer sequence in guide RNA (gRNA) since one becomes predetermined by the location of the PAM in an amplicon sequence. To overcome the PAM problem, it has been suggested to diversify PAM sequences by employing engineered Cas12a variants [6,7]. However, such variants may not be easily available to a researcher interested in the design of an amplification-based Cas12a biosensing system. In another approach, PAM sequences were introduced into amplicons with specially designed primers (e.g., [8,9]). This requires a modification of primers that can, in general, worsen their performance in amplification reaction. Furthermore, in both approaches, PAM location still predefines the sequence of the gRNA spacer, making the design of amplification-based Cas12a biosensing systems quite inflexible.

The conceptually different strategy to solve the PAM problem is to switch from double-stranded DNA (dsDNA) to single-stranded DNA (ssDNA) as a target recognized by a Cas12a/gRNA complex. It exploits the ability of Cas12a nuclease to be activated by hybridization of a gRNA spacer with a single-stranded protospacer in the absence of a PAM. Over the past two years, this strategy has been successfully incorporated into the design of amplification-based Cas12a biosensing systems [10,11,12,13,14,15]. Initially, it was realized by coupling PAM-independent Cas12a analysis of amplification products with RPA [10,11,12] and, later, with LAMP [13]. Very recently, this approach was used to combine end-point PCR and the post-PCR Cas12a-based analysis in a PAM-independent manner [14,15]. In the case of LAMP, the PAM-free Cas12a detection relied on targeting ssDNA loops which are a part of regular LAMP amplicons [13]. In contrast, DNA amplicons produced in RPA or PCR are supposed to be entirely double-stranded. Consequently, the coupling of Cas12a to RPA or PCR for PAM-free detection has required a particular mode of amplification, known as asymmetric amplification [10,11,12,14,15]. In asymmetric amplification, primer concentrations are taken unequally so that the ssDNA amplicons are generated along with the common dsDNA amplicons [16,17].

The present study aims at further development of the biosensing platform based on asymmetric PCR (aPCR) and PAM-independent Cas12a analysis of single-stranded amplicons, originally suggested in [14], by adapting it to the requirements of on-site NAT. The requirements likely include the option for a non-instrumental, visual readout of the Cas12a analysis. It is an open question whether the yield of single-stranded amplicons in an aPCR could be made sufficient to produce a visible color change in a Cas12a assay within a reasonable timeframe. Another issue is that the Cas12a nuclease does not tolerate the high temperatures used in PCR and, consequently, the aPCR/Cas12a biosensing system cannot be designed as a one-pot assay. By opening a reaction tube to transfer an aliquot of the completed aPCR to another reaction tube for downstream Cas12a analysis, the equipment and work area can be contaminated with amplicons. A mechanism to eliminate or minimize false positives caused by such contamination should be incorporated into the design of an aPCR/Cas12a biosensing system. This is thought to be especially important for on-site NAT, since such testing assumes no highly trained technical personnel are involved and the work area cannot necessarily be zoned.

Species of the *Pectobacterium* genus and a set of primers Y1 and Y2, developed by Darrasse et al. against *Pectobacterium* spp. [18], have been selected as a convenient model to achieve the study’s aims. The selected primers target the pectate lyase-encoding gene (*pel1* gene) shared by many *Pectobacterium* spp. The genus *Pectobacterium* contains species which affect a wide range of agriculturally important plants and can cause plant diseases such as soft rot, stem rot, wilt, and blackleg [19]. Agricultural plants affected by *Pectobacterium* spp. include potato, carrot, leafy greens, tomato, pepper, cucumber, and some others, where potato is by far the most important crop. Potato is ranked as the third globally consumed staple food and recommended as a food security crop by the UN Food and Agriculture Organization [20]. Blackleg and soft rot are known to result in significant losses of potato harvest both in the field and during potato storage [21]. Among *Pectobacterium* species, the present study has focused on *Pectobacterium polaris*, a newly emerging pathogenic species of the *Pectobacterium* genus, first classified in 2017 [22]. To date, the species is found in different regions of the world [22,23,24,25,26,27]. In potato, *P. polaris* causes symptoms such as blackleg, stem soft rot, and tuber decay. Also, it can potentially infect and cause diseases in a wide range of other agricultural plants such as red cabbage, broccoli, cauliflower, onion, garlic, carrot, tomato, pepper, eggplant, cucumber, squash, melon, watermelon, lettuce, sunflower and sugar beet plants [28]. Presently, molecular detection of *P. polaris* is only possible by using polymerase chain reaction (PCR), with two sets of primers. One of them is for the end-point PCR detection and provides a limit of detection (LOD) of 1 pg of *P. polaris* genomic DNA per PCR tube, whereas the other is for real-time PCR detection with a LOD of 100 pg of genomic DNA per PCR tube [27].

The study demonstrates that (i) in the absence of the PAM requirement, a strain of *P. polaris* can be reliably distinguished from strains of other *Pectobacterium* species by fine-tuning the target position of a gRNA spacer on ssDNA amplicons generated with a genus-specific primer set; (ii) the testing can be performed via both instrumental and visual Cas12a-based detection of specific single-stranded amplicons; and (iii) uracil-containing ssDNA amplicons, generated by aPCR in the presence of uracil deoxynucleotide triphosphates (dUTP), activate *Francisella tularensis* Cas12a nuclease to a level sufficient for efficient visual detection. Although *P. polaris* and other *Pectobacterium* species were used as model pathogens, the potential findings of this study could provide a foundation for developing next-generation assays for on-site detection of these pathogens.

## 2. Materials and Methods

### 2.1. Bacteria Culturing and DNA Isolation

Strains from the genera *Pectobacterium* (9 strains; 8 species), *Dickeya* (2 strains; 2 species), *Clavibacter* (7 strains; 6 species), *Agrobacterium* (1 strain, 1 species), and *Escherichia* (1 strain, 1 species) used in the study are listed in Table S1. Except for the *Clavibacter* species and *E. coli*, bacteria were cultivated as suspensions in standard LB medium (Dia-M, Moscow, Russia) at 28 °C. *E. coli* were cultivated at 37 °C. The *Clavibacter* species were grown on Petri dishes with agar medium at 28 °C as described elsewhere [13]. DNA was extracted from bacteria with the “innuPREP Bacteria DNA Kit” (IST Innuscreen GmbH, Berlin, Germany) after their pelleting by centrifugation, following the manufacturer’s instructions for Gram-positive or Gram-negative bacteria. For extracting DNA from potato, the “SKYSuper Plant Genomic DNA” isolation kit (SkyGen, Moscow, Russia) was used. DNA was isolated from 100 mg potato samples either non-contaminated or artificially contaminated with *Pectobacterium* species. Contaminated potato samples were prepared by spiking them with 10 µL of bacterial suspension in sterile distilled water. Suspensions with different bacterial concentrations were obtained for each *Pectobacterium* strain from Table S1 by 10-fold dilutions of stock suspensions. The number of bacterial genomes in DNA extracted from contaminated potato samples was quantified by real-time PCR, using the commercial kit for *Pectobacterium* spp. detection in plant tissue (“Pectobacterium spp-PB”; Syntol, Moscow, Russia) and the standard curve kindly provided by the kit’s manufacturer. For that, the real-time PCR was conducted on a DTprime5 thermal cycler (DNA-Technology, Moscow, Russia). DNA concentrations were determined on a Qubit 4 fluorimeter (Thermo Fisher Scientific, Waltham, MA, USA) with the “Qubit dsDNA BR Assay” kit (Thermo Fisher Scientific). All DNA preparations were aliquoted right after DNA isolation and aliquots were stored at –20 °C until further use.

DNA mass concentrations were converted into genome copy numbers per unit volume, based on the average genome size of *Pectobacterium* species. The size of genomes in *Pectobacterium* species lies in the range of 4.7·10^6^ to 5.0·10^6^ bp [29] and can be taken as 4.85·10^6^ bp at average. Thus, the number of genome copies in µL, *C*_n_, was calculated as follows: *C*_n_ = *C*·*N*_A_/650·4.85·10^6^, where 650 (g/mol) is the average molecular weight of base pare (bp), 4.85·10^6^ is the average genome size in bp, *N*_A_ = 6.02·10^23^ mol^−1^ is Avogadro’s number, and *C* (g/µL) is the DNA concentration.

### 2.2. Conventional and Asymmetric PCR Procedures

To perform PCR and aPCR, the commercial PCR master mix “5X qPCRmix-HS SYBR” from Evrogen (Moscow, Russia) was used. Uracil-containing amplicons were produced by PCR or aPCR with the Evrogen’s master mix “5X qPCRmix-HS (UDG)”. In the latter case, where necessary, the EvaGreen fluorescent dye (Lumiprobe, Moscow, Russia) was added to the amplification reaction mixture at a 40-fold dilution of the commercial stock solution of the dye to monitor amplification in real time. Bacterial and/or potato DNA was introduced into PCR or aPCR by adding 1 µL of DNA solution of the desired concentration. The final volume of amplification mixture was 20 µL. Primers Y1 and Y2 [18] used to perform PCR were chemically synthesized and HPLC-purified by Lumiprobe. The sequences (Table S2) were 5′-TTACCGGACGCCGAGCTGTGGCGT-3′ for Y1 primer and 5′-CAGGAAGATGTCGTTATCGCGAGT-3′ for Y2 primer. The concentrations of primers were either 400 nM of each or the concentration of one of the primers varied from 400 nM to 40 nM while that of another was kept at 400 nM. All dilutions were made with deionized (18 MΩ) Milli-Q water. aPCR runs were performed on a portable YourPCR thermocycler (YourPCR, Moscow, Russia) controlled via a smartphone application, operating in an end-point detection mode. Real-time PCR or aPCR, as well as melting curve analysis, were carried out on a Bio-Rad CFX96 thermal cycler (Bio-Rad Laboratories, Hercules, CA, USA). Unless otherwise indicated, amplification runs were started with 10 min pre-heat at 95 °C, followed by 30 to 60 cycles with 1 min at 95 °C, 1 min at 65 °C, and 1.5 min at 72 °C and finished with 3 min final extension at 72 °C. The amplification runs were conducted in triplicate if not stated differently. Where required, PCR samples were subjected to heating for 5 min at 95 °C post-PCR and then placed in an ice water bath for sharp cooling. To verify the sequence of the amplified segment of bacterial genomic DNA, amplicons from *Pectobacterium* species were subjected to Sanger sequencing carried out by Evrogen. Corresponding sequences retrieved from the NCBI databases and those obtained by the Sanger sequencing are provided in Table S3.

### 2.3. Cas12a Cleavage Assay

To perform the Cas12a-based analysis of ssDNA amplicons, the recombinant CRISPR-nuclease Cas12a from *Francisella tularensis* was heterologously expressed and purified as described elsewhere [30]. gRNAs (Table 1) were produced enzymatically, using “TranscriptAid T7 High Yield Transcription Kit” (Thermo Fisher Scientific). To produce gRNAs, one of the templates for gRNA synthesis and the oligonucleotide T7P, listed in Table S2, were mixed at equimolar concentrations. gRNAs were purified as described in [13]. If not stated otherwise, Cas12a nuclease and gRNA (each at the final concentration of 60 nM) were mixed in the reaction buffer composed of 40 mM Tris-HCl, 40 mM glycine, 6 mM MgCl_2_, 1 mM dithiothreitol, 0.001% Triton X-100, and 0.4% polyethylene glycol (pH 8.6) and left on a bench for 10 min. As a rule, 19 µL of the prepared Cas12a/gRNA mixture was combined with 1 µL of a solution of “molecular reporters” (MRs) so that the final MR concentration was 1 µM. The MR sequence is provided in Table S2. MR is a short DNA oligonucleotide labeled with FAM (6-carboxyfluorescein) and BHQ-1 (fluorescence quencher) at its termini. Immediately thereafter, a 1 µL aliquot of completed aPCR was added if not indicated otherwise. For control, the same volume of PCR no-template control (PCR NTC) was used. The prepared Cas12a reaction mixtures were used immediately for fluorescence measurements. The real-time monitoring of Cas12a *trans*-cleavage activity was carried out on a DTprime5 thermal cycler at a constant temperature of 37 °C. The fluorescence intensity was measured in 1 min intervals. For visual detection, the test tubes were placed either on a blue light transilluminator SkyLight-Pad Edge 20-blue V1 (Viber, Eberhardzell, Germany) or illuminated with a Blue Light LED flashlight (TaoYuan Optoelectronics Shenzhen Co., Shenzhen, China) against a dark background. The test tube images were captured with a smartphone camera through an orange filter.

### 2.4. Statistical Treatment

Arithmetic means, standard deviations, and confidence level *p*-values were calculated using Microsoft Excel’s statistical functions (the two-tailed Student’s *t*-test), based on the results of 3 to 5 independent experiments.

## 3. Results

### 3.1. The Performance of the Y1–Y2 Primer Pair in Conventional PCR

Firstly, the performance of the Y1–Y2 primer set (Table S2) was evaluated in conventional, symmetric PCR (at the primer molar ratio of 1:1). The originally suggested duration times of 60 s, 60 s, and 90 s for denaturation, annealing, and extension steps, respectively [18], were used (the PCR regime is hereafter referred to as “60–60–90”). This step was undertaken to establish “reference points” for the conversion of conventional PCR into aPCR and its further optimization. For the sake of convenience, real-time PCR was employed to evaluate the primers’ performance.

The primers Y1 and Y2 generated PCR products for all strains of *Pectobacterium* species used in the study (Figure S1). However, they also generated products in control test tubes with no bacterial DNA (PCR NTCs) in the late cycles of a 40-cycle run (Figure S1). The melting curve analysis showed that amplicons produced in PCR NTC have melting temperatures (*T*_m_) noticeably lower than the *T*_m_ of amplicons produced in PCRs with the DNA of *Pectobacterium* species (81 °C vs. 87 °C, Figure S2). Since the size of amplicons is 435 base pairs (bp) (Table S3), the high melting temperature of 87 °C is expected for specific products. One may suggest that non-specific amplicons with a *T*_m_ of 81 °C are produced in PCR NTCs in the late PCR cycles, likely due to the susceptibility of Y1–Y2 primers to form primer-dimers. This suggestion was supported by the results of electrophoretic analysis (Figure S3a). The DNA fragments matching the expected size of 435 bp were generated in the presence of bacterial DNA, while DNA fragments of about 60 bp in length were observed for PCR NTC. Interesting, these unspecific amplicons have rather a high *T*_m_ value of 81 °C for such a size (Figures S2 and S3). The reason behind it may be the high GC content of primer Y1 (66%, Table S2).

The detection sensitivity of conventional PCR with the Y1–Y2 primer pair was evaluated with genomic DNA of *P. polaris*, *P. carotovorum*, and *P. brasilience*. The series of 10-fold dilutions of genomic DNA were prepared so that the number of copies of bacterial genomes varied from 10^6^ copies down to one copy per µL of solution. The concentration dependences of quantitation cycle (*C*_q_, provided by PCR thermocycler software CFX Manager 3.0) on the number of genome copies in a PCR tube were linear on a semi-logarithmic scale (Figure S4). It should be noted that for a load of one genome copy, only *C*_q_ values corresponding to specific PCR products (as judged by the melting curve analysis) have been taken into consideration. If melting curve analysis demonstrated that products may be unspecific (*T*_m_ is around of 81 °C compared to that of about 87 °C for specific products, Figure S2) or represent a mixture of specific and unspecific amplicons (as exemplified in Figure S5), such *C*_q_ values have been excluded. For loads of 10 genome copies or above, merely specific products (*T*_m_ ≈ 87 °C) were observed. Thus, finally, the LOD of 10 genome copies per PCR may be taken as a “reference value” for detection of *Pectobacterium* species by conventional PCR with the Y1–Y2 primer pair.

### 3.2. Asymmetric PCR with the Y1–Y2 Primer Pair and Detection of Single-Stranded Amplicons with a Cas12a/gRNA Complex

The intrinsic sensitivity of amplification-free Cas12a detection is known to basically fall into a picomolar range [31]. Thus, achieving a high overall sensitivity for the aPCR/Cas12a biosensing system would depend solely on the aPCR step. In aPCR, the production of single-stranded amplicons relies on the presence of double-stranded amplicons, which serve as templates for their generation in a linear fashion [16,17]. Since aPCR is less efficient at producing double-stranded amplicons than conventional PCR, an aPCR run may need to take significantly longer than 40 cycles.

To convert conventional PCR with the Y1–Y2 primer pair into aPCR, an arbitrary primer molar ratio of Y1/Y2 = 2 has been taken at the first step. With the original cycle duration time (the PCR regime “60–60–90” [18]), the overall time of aPCR was about 3 h for 40 cycles and almost 4 h for 60 cycles. To decrease the aPCR run time, the durations of cycle steps (denaturation, annealing, and extension) were varied as shown in Table 2.

**Figure 1:**
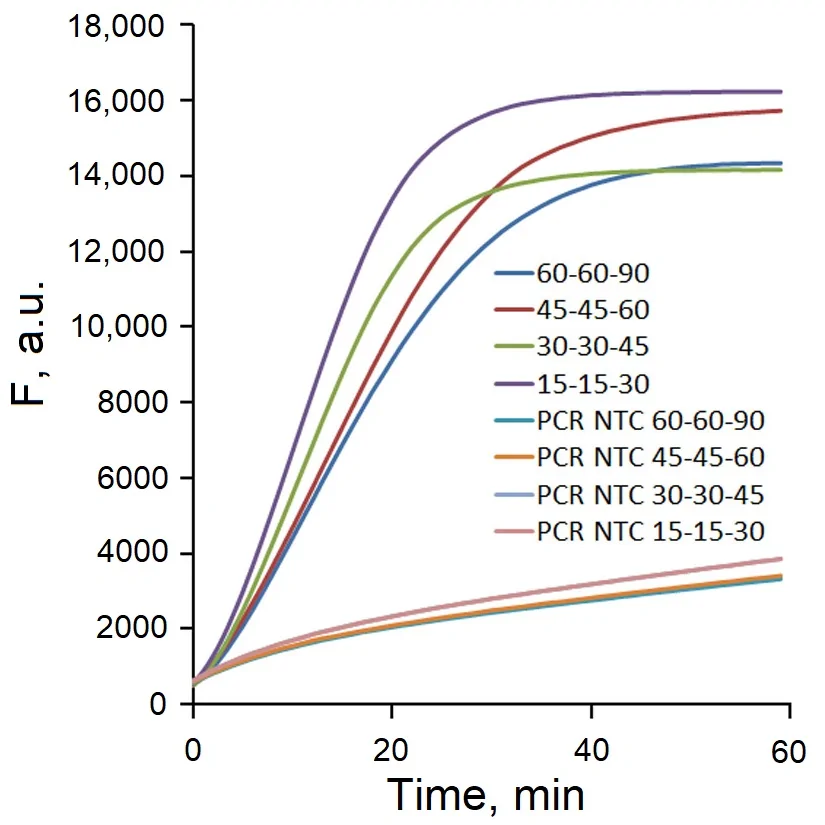
Activation of Cas12a nuclease by aPCR amplicons produced with the Y1/Y2 molar ratio of 2. Representative kinetic curves for MR cleavage. gRNA1. An amount of 1 µL of the completed PCR or PCR NTC per 20 µL of Cas12a reaction mixture, 37 °C. aPCR with genomic DNA of *P. polaris*, 200 genome copies per aPCR. aPCR regimes are indicated in the figure as in Table 2. F—fluorescence in arbitrary units (a.u.).

As seen, for the case of *P. polaris*, *C*_q_ values increased when the cycle duration time decreased. However, the overall time of an aPCR run with 60 cycles has become markedly lesser for the PCR regime “15–15–30” (the durations of denaturation, annealing, and extension steps are 15 s, 15 s, and 30 s, respectively). The aPCR products generated after 60 cycles were tested using a complex of Cas12a nuclease with gRNA1 (Table 1). The gRNA1 spacer was designed so as to target a segment in single-stranded amplicons, which are expected to be produced by the twofold excess of the Y1 primer. The sequence of this segment was identical for the *Pectobacterium* species used in the study (nucleotide positions from 252 through 271, Table S3). As shown by Figure 1, the aPCR amplicons generated at the Y1/Y2 molar ratio of 2 did induce the Cas12a activation, as exemplified with PCR products from genomic DNA of *P. polaris*. Since gRNA1 was designed to target a segment in dsDNA amplicons with no corresponding PAM (Table S3), this evidently indicates that ssDNA amplicons can be produced in aPCR with the twofold excess of Y1 primer and that such single-stranded amplicons do not require PAM to activate Cas12a nuclease.

The variations in the cycle duration time appeared not to affect the production of target ssDNA amplicons to a substantial extent since the kinetics of MR cleavage by Cas12a nuclease was approximately the same (Figure 1). In fact, as may be judged by the cleavage kinetics, the yield of target ssDNA amplicons for the shortest cycle duration time can even be somewhat higher. So, the PCR regime “15–15–30” has been selected for further experiments. For this PCR regime, the primer molar ratio was further varied in the range of 1 to 20. Either primer Y1 (the Y1/Y2 ratio) or primer Y2 (the Y2/Y1 ratio) was present in excess to favor synthesis of target or non-target DNA strands, respectively. Furthermore, PCR samples were either subjected or not subjected to the post-PCR heat treatment (the heating/cooling step). PCR products in all PCR samples were tested with the Cas12a/gRNA1 complex.

**Figure 2:**
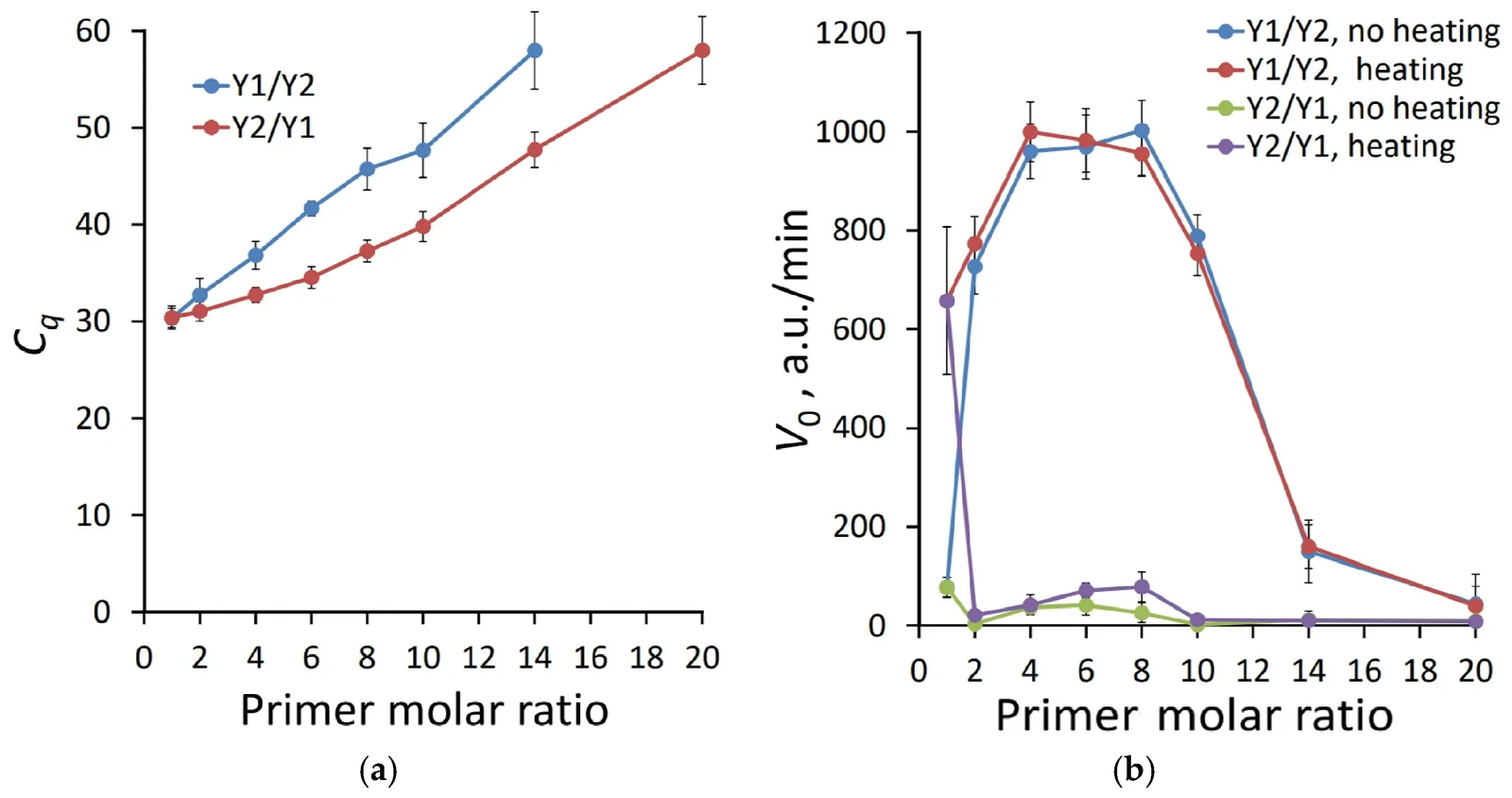
The impact of primer ratio on aPCR performance and the yield of ssDNA amplicons. PCR regime—“15–15–30”. The load of *P. polaris* DNA—100 genome copies per PCR. The mean values and the corresponding standard deviations are shown. (**a**) Dependence of the quantification cycle, *C*_q_, on the molar ratio of Y1 and Y2 primers. (**b**) Dependence of the initial rate of MR cleavage by the activated Cas12a nuclease, *V*_0_, on the molar ratio of Y1 and Y2 primers. The *V*_0_ values were obtained by subtracting the corresponding values for PCR NTCs. An amount of 1 µL of PCR per 20 µL of Cas12a reaction mixture, 37 °C.

The results of systematic variations in the primer molar ratio are presented in Figure 2 for the case of *P. polaris*. Amplification efficiency for dsDNA amplicon production declines when the concentration of one of the primers decreases, as may be judged by comparing *C*_q_ values (Figure 2a, the representative amplification curves are provided in Figure S6). The decline was more pronounced with the excess of Y1 primer and at higher primer molar ratios. At the Y1/Y2 molar ratio of 20, the *C*_q_ values were beyond the 60-cycle run. At the same time, the yield of target ssDNA amplicons negligible at the equimolar primer concentrations has been greatly enhanced at the Y1/Y2 molar ratios of 2 to 10 (Figure 2b). The yield was estimated by comparing the Cas12a activation, based on the initial rate of MR cleavage, *V*_0_. Here, *V*_0_ is a slope of the near-linear increase in fluorescence at the beginning of a kinetic curve, as illustrated by Figure S7. The *V*_0_ values reached a plateau at the Y1/Y2 molar ratios of 4 to 8 and started to decline at the ratio of 10. No appreciable activation of Cas12a nuclease was observed at the Y1/Y2 molar ratios of 14 and 20 (Figure 2b). If PCR products were subjected to the post-PCR heat treatment, the marked Cas12a activation was already observed at a Y1/Y2 molar ratio of 1. For the higher Y1/Y2 molar ratios, the *trans*-cleavage activities of Cas12a were statistically indistinguishable between the heat-treated and untreated samples (Figure 2b). The expected excessive production of the non-target DNA strand in aPCR with the Y2/Y1 primer molar ratios ≥ 1 resulted in PCR products unable to activate Cas12a nuclease, irrespectively of the postPCR heat treatment.

Although some peaking of mean *V*_0_ values is observed between the Y2/Y1 molar ratios from 2 to 10, these mean values do not differ statistically significantly (*p*-values ≥ 0.05). It should also be noted that activation of Cas12a nuclease by amplicons generated in PCR at the Y1/Y2 molar ratio of 1 and, subjected to the post-PCR heat treatment, resulted in a greater scattering of *V*_0_ values (Figure 2b). Thus, as a reasonable compromise between the yield of single-stranded amplicons and the duration of aPCR, the Y1/Y2 molar ratio of 4 has been chosen for further experiments.

To ensure additionally that specific amplicons are produced under conditions of aPCR with the Y1/Y2 molar ratio of 4, aPCR products were subjected to electrophoretic analysis. The specific dsDNA amplicons were predominantly generated in aPCR at the load of 10 copies of *P. polaris* genome per reaction (Figure S3). Yet, some number of unspecific amplicons of about 60 bp in length as that in NTC PCR was also observed.

### 3.3. Sensitivity of aPCR/Cas12a Detection System

**Figure 3:**
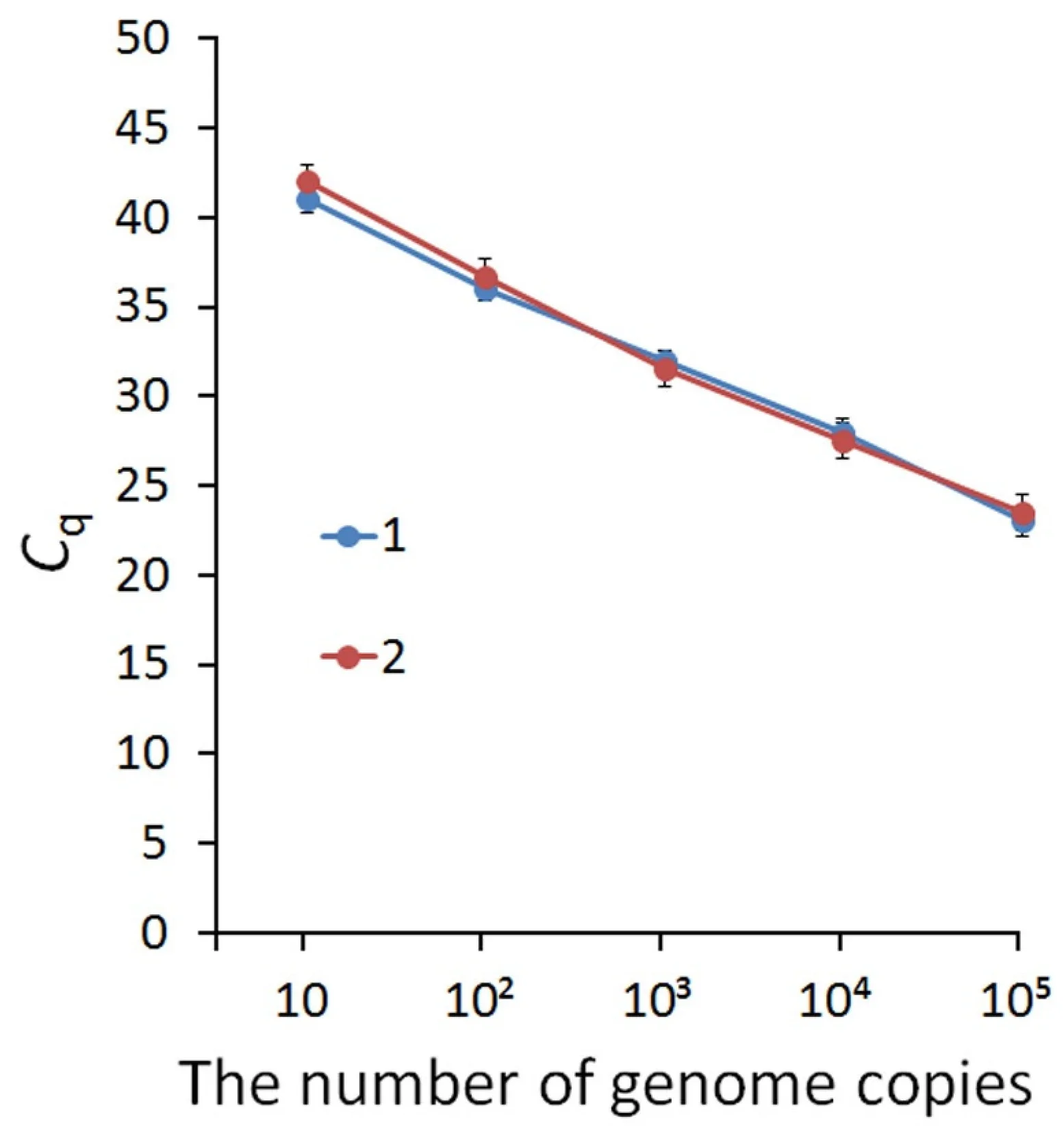
The detection of *P. polaris* with the optimized aPCR. Dependences of the quantification cycle, *C*_q_, on the number of bacterial genomes per PCR reaction: 1—bacterial DNA alone; 2—bacterial DNA in the presence of 100 ng of potato DNA. The mean values and corresponding standard deviations are shown.

The sensitivity of the optimized aPCR (the regime “15–15–30”, Y1/Y2 = 4) has been determined for bacterial DNA alone and in the presence of 100 ng of potato DNA, using *P. polaris* as an example. As seen from Figure 3, the dependencies of *C*_q_ on DNA loads were linear in a semi-logarithmic scale in both cases and practically coincided. The performance of the optimized aPCR with regard to generation of specific and non-specific amplicons at loads of one copy of bacterial genome per PCR tube and for PCR NTC was similar to that observed earlier for PCR with the regime “60–60–90” and the Y1/Y2 molar ratio of 1 (as illustrated by Figures S2 and S5). Therefore, the value of LOD for the optimized aPCR was not compromised compared to that of conventional PCR with the Y1–Y2 primer pair and may be taken as 10 copies of bacterial genome per PCR as well. For all loads of *P. polaris* DNA (Figure 3), significant activation of Cas12a nuclease by amplification products was observed (Figure S8).

**Figure 4:**
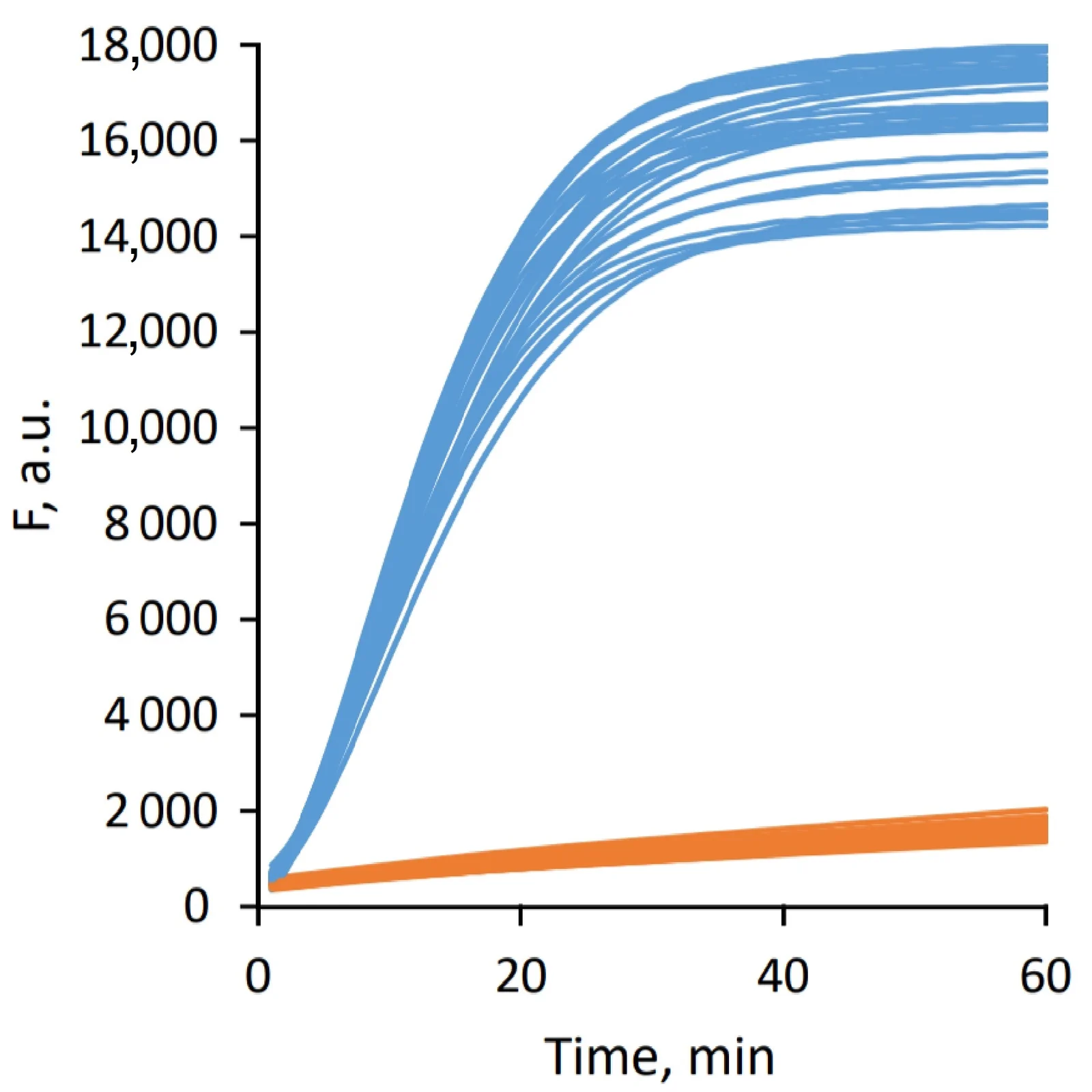
The fluorescence kinetic curves reflecting the MR cleavage by activated Cas12a nuclease. Thirty two aPCR runs (PCR regime—“15–15–60”, Y1/Y2 molar ratio = 4) conducted on the YourPCR thermocycler operating in end-point mode. aPCR products analyzed with the Cas12a/gRNA1 complex. An amount of 1 µL of the completed aPCR per 20 µL of the Cas12a reaction mixture, 37 °C. Blue and orange curves correspond to aPCR conducted with 10 copies of *P. polaris* genome per reaction and to PCR NTCs, respectively. F—fluorescence in arbitrary units (a.u.).

To validate this LOD value, we have conducted 32 runs of the optimized aPCR with the load of 10 copies of *P. polaris* genome and the corresponding PCR NTCs. The runs were conducted on the YourPCR thermocycler operating in end-point mode. The aPCR products were tested with the Cas12a/gRNA1 complex, and the results are provided in Figure 4 as fluorescence kinetics curves. In all cases, the well-defined fluorescence kinetic curves were observed, which clearly differed from the fluorescence responses for PCR NTCs. One may conclude that overall sensitivity of the aPCR/Cas12a detection system is determined by the sensitivity of aPCR and may also be taken as 10 copies of bacterial genomes per aPCR.

### 3.4. Selectivity of aPCR/Cas12a Detection System Toward Pectobacterium Species

The selectivity of the optimized aPCR in conjunction with Cas12a analysis of aPCR products has been evaluated with the strains listed in Table S1 and gRNA1. At the load of 10 genome copies per PCR, all *Pectobacterium* strains from Table S1 were identified by a subsequent Cas12a analysis of amplicons as belonging to genus *Pectobacterium* (Figure S9). For non-*Pectobacterium* species, some PCR products were also generated in the optimized aPCR as manifested by the occurrence of *S*-shaped amplification curves (Figure S10), though at a rather high load of bacterial DNA of 0.5 ng. Yet, these products were not recognized by the Cas12a/gRNA1 complex as target amplicons. The corresponding fluorescence kinetic curves were indistinguishable from those obtained for PCR NTCs (Figure S9). Thus, the other species from Table S1, belonging to the genera *Dickeya*, *Clavibacter*, *Agrobacterium*, and *Escherichia*, are not identified by the Cas12/gRNA1 analysis of aPCR products as *Pectobacterium* species.

### 3.5. The Selective Identification of P. Polaris Among Pectobacterium Species with the aPCR/Cas12a System

To selectively detect a strain of *P. polaris*, three gRNA variants (gRNA2, gRNA3, and gRNA4, Table 1) have been designed to target the 27-nucleotide long segment of ssDNA amplicons whose sequence was not identical among the *Pectobacterium* spp. used in the study. Compared to the sequence of *P. polaris*, this segment (nucleotides from 78 through 104 in the amplicon sequence alignment in Table S3) contained two to six nucleotide substitutions for other *Pectobacterium* species studied, according to their sequences retrieved from NCBI databases. The presence of substitutions was confirmed by Sanger sequencing, except for *P. brasiliense*. For the *P. brasiliense* strains used in the study, the additional nucleotide substitution in position 90 was found compared to the retrieved sequence (Table S3). Within this 27-nucleotide segment, 20-nucleotide spacers of the designed gRNAs align with 3–4 nucleotide shifts, so as to have three to six mismatched nucleotides for *Pectobacterium* species other than *P. polaris* (Figure S11).

**Figure 5:**
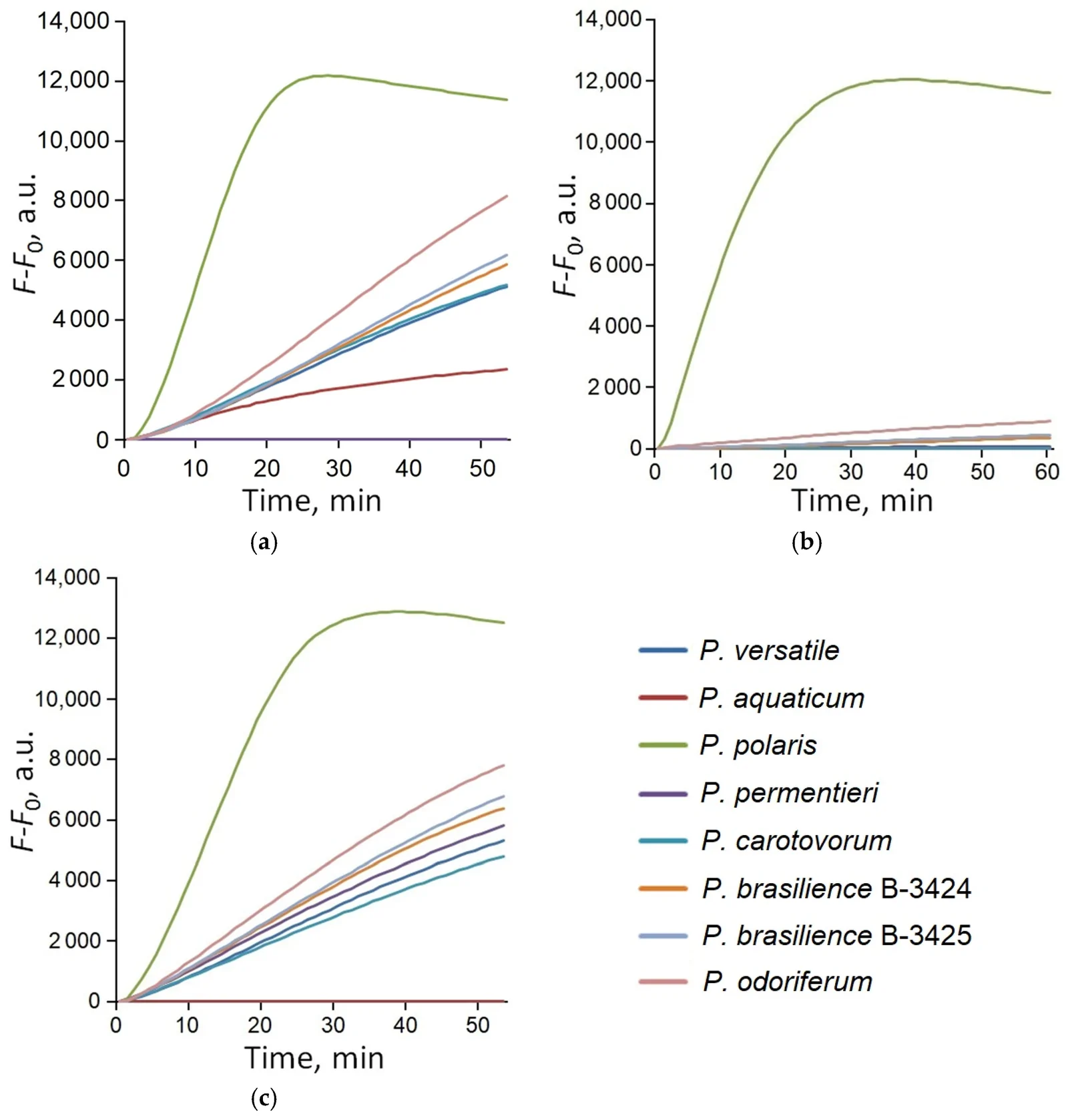
Representative kinetic curves for MR cleavage by Cas12a nuclease complexed with gRNA2 (**a**), gRNA3 (**b**), and gRNA4 (**c**). An amount of 1 µL of the completed aPCR per 20 µL of Cas12a reaction mixture, 37 °C. The optimized aPCR (“15–15–30”, Y1/Y2 = 4). A total of 100 copies of bacterial genomes per PCR. The *Pectobacterium* species tested are indicated in the figure. *F* and *F*_0_—fluorescence with 1 µL of completed aPCR and PCR NTC, respectively.

**Figure 6:**
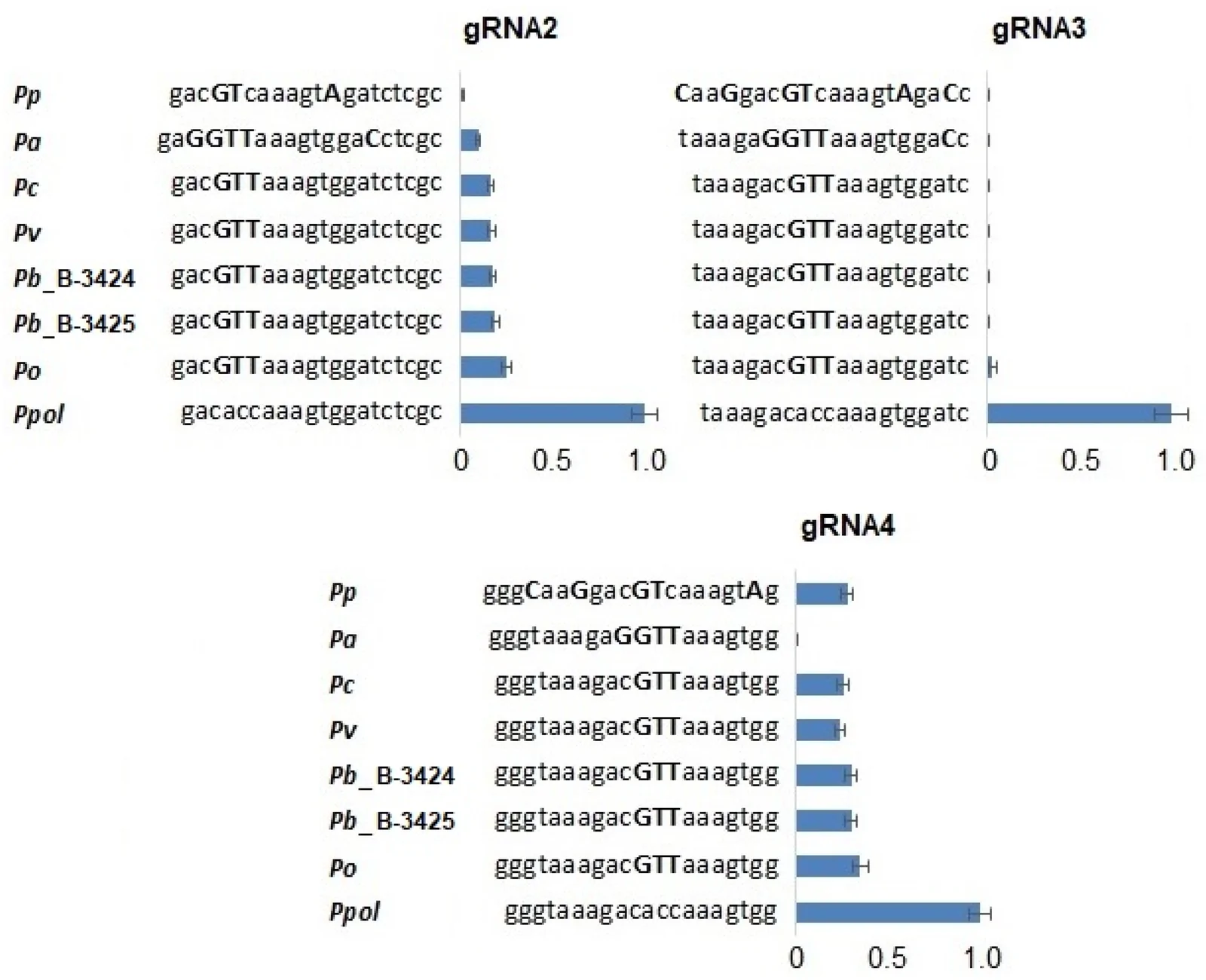
Impact of mismatches on cleavage kinetics. Histograms representing the relative mean values of *V*_0_ for various *Pectobacterium* strains and gRNA2, gRNA 3, and gRNA4. The mean *V*_0_ values for *P. polaris* are taken as unity. Each bar is accompanied by a relevant protospacer sequence with mismatches indicated by capital bold letters. *Pp*, *Pa*, *Pc*, *Pv*, *Pb*_B-3424, *Pb*_B-3425, *Po*, and *Ppol* stand for *P. parmantieri*, *P. aquaticum*, *P. carotovorum*, *P. versatile*, *P. brasiliense* strain B-3424, *P. brasiliense* strain B-3425, *P. odoriferum*, and *P. polaris*, respectively. The mean values and corresponding standard deviations are shown. Other conditions are as in Figure 5.

Figure 5 shows representative curves for cleavage kinetics for complexes of Cas12a nuclease with gRNA2, gRNA3, and gRNA4. The Cas12a nuclease was activated by ssDNA amplicons generated in the optimized aPCR for different *Pectobacterium* species. Clearly, gRNA3 demonstrates the best selectivity toward *P. polaris* amplicons. The relative values of *V*_0_ for each *Pectobacterium* strain and gRNA are also presented as histograms in Figure 6. The relative *V*_0_ values were calculated for a given gRNA by taking the mean *V*_0_ value for *P. polaris* as unity. Each histogram bar is accompanied by the sequence of relevant protospacers with mismatches indicated with bold capital letters.

Statistically, *P. polaris* differed significantly (*p*-values < 0.0001) by the mean *V*_0_ values from other *Pectobacterium* species for any of these gRNAs. As seen, there is a single three-nucleotide mismatch (GTT) in the gRNA/DNA heteroduplexes for species *P. odoriferum*, *P. brasiliensis*, *P. versatile*, and *P. carotovorum* (Figure 6). For these species, mean *V*_0_ values declined by 75–83% for gRNA2 and by 65–78% for gRNA4, but their absolute values were still appreciable (Figure 5a,c). The mean *V*_0_ values were statistically indistinguishable (*p*-values > 0.05) among these four species for each gRNA. In contrast, the same mismatch resulted in practically no MR cleavage for gRNA3 (Figure 5b and Figure 6). Clearly, the position of such a mismatch may be of decisive importance for the Cas12a nuclease activation. Yet, it could also be a complex interplay between a spacer sequence, per se, and the relative position of a mismatch (or mismatches in general). For instance, in *P. parmantieri*, the three-nucleotide mismatch GTT reduces to the two-nucleotide mismatch GT. In the case of gRNA2, this mismatch is accompanied by an additional single-nucleotide mismatch, while for gRNA3 and gRNA4 it is accompanied by four or three additional single-nucleotide mismatches, respectively (Figure 6). Despite the higher overall number of mismatches for gRNA4 than that for gRNA2, the Cas12a/gRNA4 complex can nonetheless acquire a noticeable *trans*-cleavage activity (Figure 5 and Figure 6). The corresponding mean *V*_0_ value differs statistically, with *p*-value less than 0.0002. For another *Pectobacterium* species, *P. aquaticum*, the four-nucleotide mismatch has resulted in a practically complete absence of the Cas12a *trans*-activity for gRNA4. However, the occurrence of the additional single-nucleotide mismatch (dT → dC) in the case of gRNA3 resulted in an observable *trans*-activity of Cas12a nuclease (Figure 6).

To demonstrate the applicability of the aPCR/Cas12a system with gRNA3 for the detection of *P. polaris* in potato tuber tissue, DNA was extracted from the artificially contaminated potato samples and examined with both the commercial real-time PCR assay against *Pectobacterium* spp. and the developed detection system. The PCR results were concordant with those obtained with the PAM-independent aPCR/Cas12a detection system (Table S4).

### 3.6. The Recognition of Uracil-Containing ssDNA Amplicons by a Cas12a/gRNA Complex

In conventional PCR, the introduction of uracil into DNA amplicons is routinely employed in laboratory settings to prevent false-positive results due to carryover contamination [32]. To evaluate whether and how the efficiencies of aPCR and subsequent Cas12a analysis may be affected by the uracil introduction, the commercial PCR master mix with 50% replacement of deoxythymidine-5′-triphosphate (dTTP) with dUTP was used. The performance of the optimized aPCR with 50% substitution of dTTP by dUTP was found to worsen: the *C*_q_ values increased by about four to five cycles on average. This may be expected since, for conventional PCR, the amplification efficiency is known to decrease 10% to 25% in the presence of dUTP, depending on the dUTP percentage and the specific enzyme used [32].

**Figure 7:**
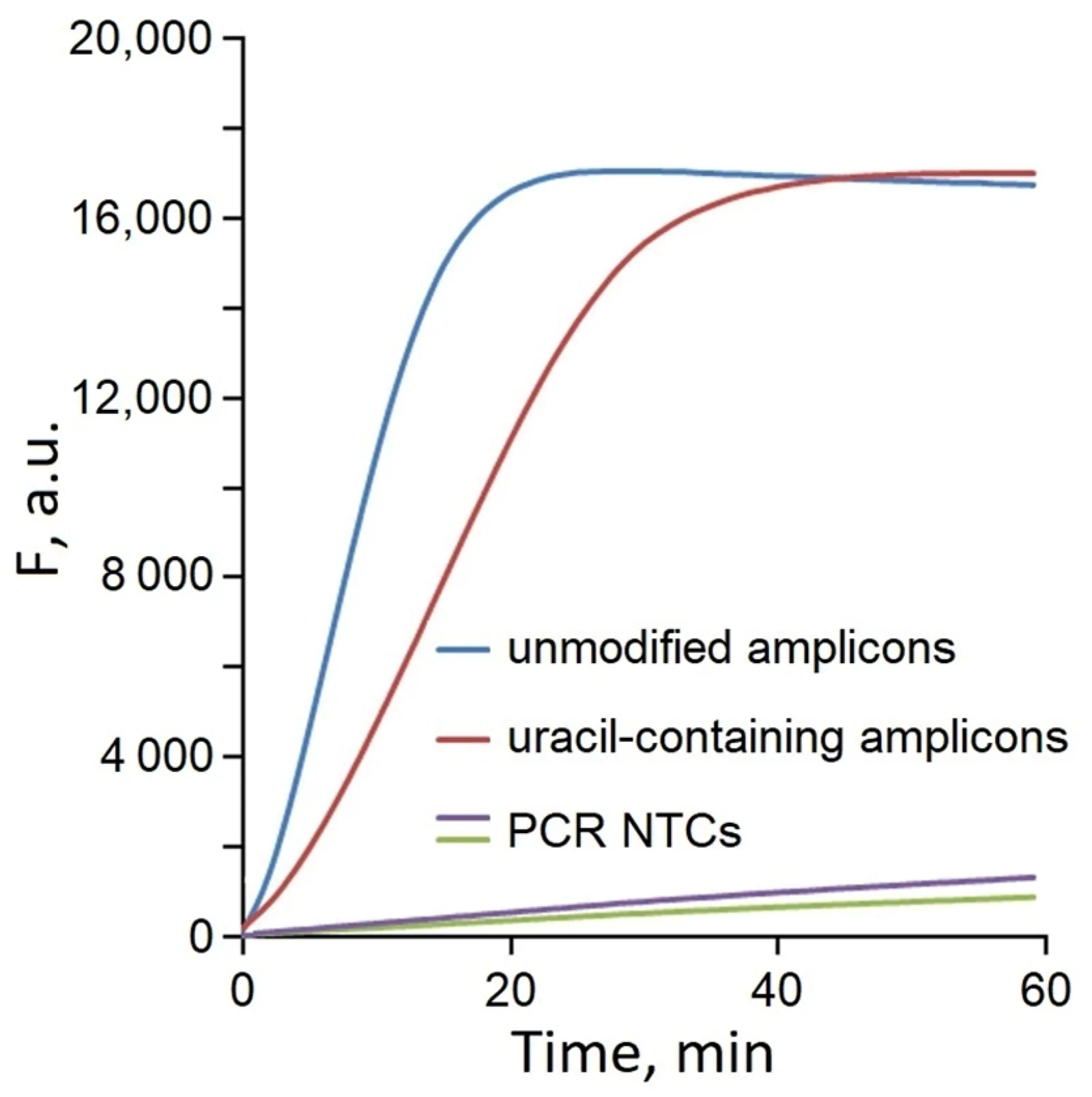
Representative kinetic curves for MR cleavage by Cas12a nuclease activated by unmodified and uracil-containing PCR amplicons. gRNA3. An amount of 1 µL of the completed aPCR per 20 µL of Cas12a reaction mixture, 37 °C; an elevated magnesium concentration of 18 mM. The optimized aPCR (“15–15–30”, Y1/Y2 = 4); 10 copies of *P. polaris* genomes per PCR.

The uracil-containing ssDNA amplicons generated in the optimized aPCR were found to activate Cas12a complexed with gRNA3 (Figure S12). Nevertheless, the cleavage kinetics were noticeably slower than those with unmodified ssDNA amplicons (Figure S12 vs. Figure 5b). To increase the cleavage rate, the magnesium concentration in the Cas12a reaction mixture was varied in the range of 6 to 30 mM. Earlier, the increase in magnesium concentration in the Cas12a reaction mixture allowed the MR cleavage in the Cas12a analysis of LAMP products to be boosted [13]. Indeed, the increase in magnesium concentration to 18 mM sped up the cleavage rate, followed by a decline in the rate at higher magnesium concentrations (Figure S13). Figure 7 shows the kinetics of MR cleavage by Cas12a nuclease with 18 mM of magnesium after addition of uracil-containing PCR products. The kinetics became comparable with that of unmodified PCR products at 6 mM of magnesium under otherwise identical conditions (Figure 7 vs. Figure 5b).

It is possible that the MR cleavage rate may also be accelerated by increasing the number of uracil-containing ssDNA amplicons in a Cas12a reaction mixture. The simplest way to do it is by adding a larger volume of a completed aPCR to a Cas12a assay. However, no increase in the cleavage rate was observed with the added volume (Figure S14). Moreover, when 10 µL of aPCR was added, the cleavage rate clearly started to decline. It is thought that the expected positive effect of a larger amount of target DNA can be offset by the negative impact of introducing more PCR buffer components and/or aPCR byproducts into the Cas12a reaction.

### 3.7. The Visual Detection Mode

**Figure 8:**
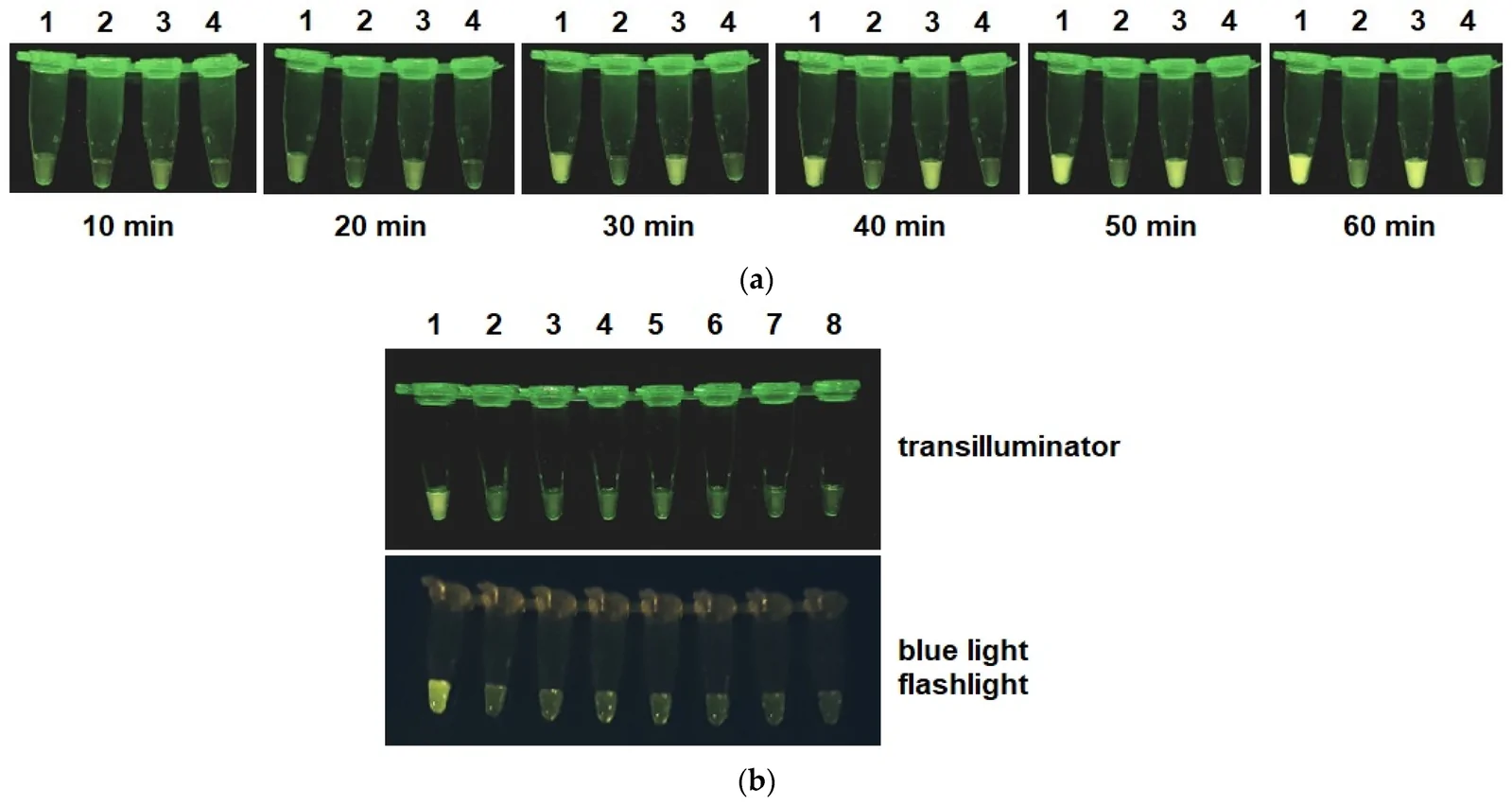
The visual analysis of MR cleavage by the *trans*-activated Cas12a nuclease. Activation by uracil-modified ssDNA amplicons, 18 mM magnesium in the Cas12a reaction. (**a**) The time course of yellowish color development after the addition of aPCR products: 1 and 3—the optimized aPCR with 10 genome copies of *P. polaris*; 2 and 4—the corresponding PCR NTCs. The addition of 1 µL (tubes 1 and 2) or 5 µL (tubes 3 and 4) aliquots of aPCRs. (**b**) Various *Pectobacterium* species, in 30 min after the addition of a 1 µL aliquot of aPCR to a Cas12a reaction mixture: 1 to 7—*P. polaris*, *P. carotovorum*, *P. parmantieri*, *P. aquaticum*, *P. versatile*, *P. odoriferum*, *P. brasliensis* strain B-2424, respectively; 8—PCR NTC. The optimized aPCR with 100 copies of bacterial genomes per reaction.

On-site NAT would benefit from visual detection, as it further simplifies the required equipment. Thus, after the optimization of Cas12a detection of uracil-containing ssDNA amplicons using instrumental analysis, the outcome of MR cleavage by activated Cas12a nuclease was evaluated by visual observation of test tubes upon their illumination with blue light. For that, 60-cycle aPCR was conducted with “5X qPCRmix-HS (UDG)” master mix (uracil-containing amplicons) with 10 genome copies of *P. polaris* DNA as a template on the Bio-Rad CFX96 PCR machine in the absence of fluorescent dye (the end-point detection mode). Runs with “5X qPCRmix-HS (UDG)” master mix included a 10 min incubation step at 37 °C prior to PCR cycling, as recommended by the manufacturer’s standard protocol. Aliquots in 1 µL or 5 µL amounts of the completed PCRs or the corresponding PCR NTCs were used for the Cas12a analysis. The reaction tubes were placed on a transilluminator equipped with an orange filter and the MR cleavage reaction proceeded at ambient temperature. The development of yellowish color was evident in 30 min after the aliquot addition (Figure 8a). At the same time, no appreciable color development was observed up to 60 min of incubation for reactions with PCR NTCs. As expected, the addition of 5 µL instead of 1 µL had no obvious advantage, likely due to the negative impact of introducing more PCR components/byproducts into the Cas12a reaction.

The Cas12a analysis with visual readout allowed to selectively discriminate *Pectobacterium* species other than *P. polaris* (exemplified by Figure 8b). We also tested the option of illuminating the reaction tubes with a blue light flashlight instead of a laboratory transilluminator. As seen from Figure 8b, the visual detection is quite possible with such a simple and inexpensive autonomous source of blue light.

**Figure 9:**
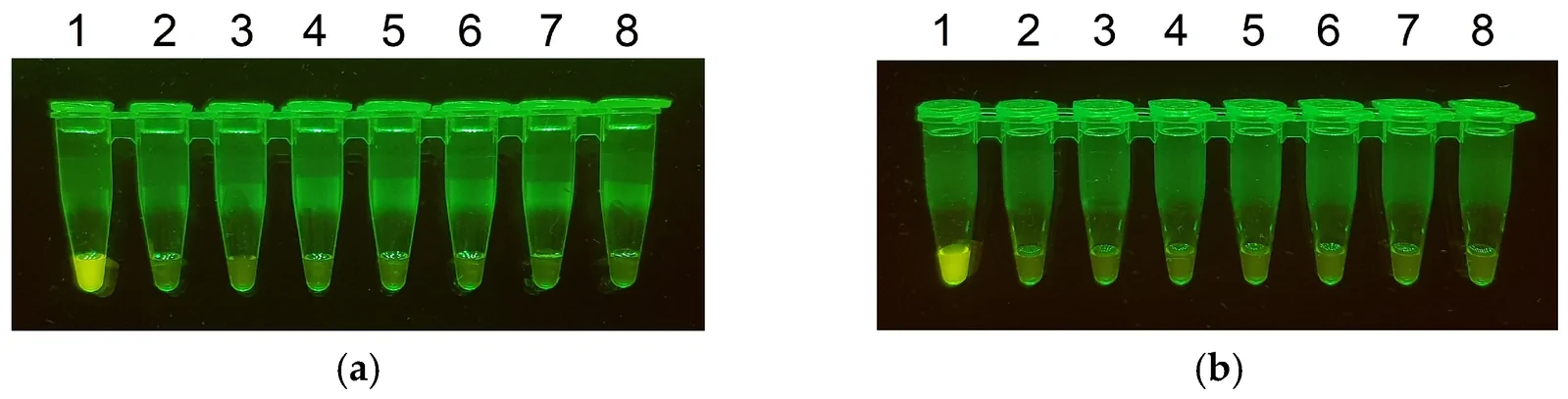
The detection of *P. polaris* by aPCR/Cas12a biosensing system with a visual readout. (**a**) 1 to 7—*P. polaris*, *P. carotovorum*, *P. parmantieri*, *P. aquaticum*, *P. versatile*, *P. odoriferum*, *P. brasliensis* strain B-2424, respectively; 8—PCR NTC. The optimized aPCR with 10 copies of *P. polaris* genome per reaction or 200 copies of bacterial genomes of other *Pectobacterium* species per reaction. All aPCRs additionally contained 200 ng of potato DNA. (**b**) Analysis of DNA extracted from potato samples artificially contaminated with *Pectobacterium* species. The optimized aPCR, 200 ng of DNA per reaction: 1 to 7—12, 230, 160, 210, 170, 175, 150 copies of *P. polaris*, *P. carotovorum*, *P. parmantieri*, *P. aquaticum*, *P. versatile*, *P. odoriferum*, *P. brasliensis* (strain B-2424) genomes per reaction, respectively; 8—PCR NTC. The number of genome copies was determined essentially as described in Table S4 for *P. polaris*. The other conditions as in Figure 8.

Similar results were obtained when the analyzed samples additionally contained 200 ng of potato DNA, or when using DNA extracted from potato samples artificially contaminated with *Pectobacterium* species (Figure 9). In both cases, the optimized 60-cycle aPCR was performed on the YourPCR thermocycler, using “5X qPCRmix-HS (UDG)” master mix. The downstream Cas12a/gRNA3 analysis of uracil-containing ssDNA amplicons was carried out with a visual readout, using the transilluminator. As seen from Figure 9, down to 10 copies of a *P. polaris* genome per reaction can be detected with the designed aPCR/Cas12a biosensing system working in a mode compatible with the requirements of on-site NAT.

## 4. Discussion

On-site NAT is commonly considered an approach based on isothermal amplification of nucleic acids. Integrating Cas12a nucleases with RPA or LAMP is an emerging trend in developing assays for on-site NAT. However, the design of efficient RPA or LAMP primers is a much more challenging task than that of PCR primers. On-site NAT could definitely benefit from integrating Cas12a nucleases with PCR into a biosensing system relying on low-cost thermocyclers and a selective but equipment-free Cas12a-based readout of the amplification outcome. This potentially allows the vast number of already verified PCR primers developed for laboratory testing to be applied to on-site NAT.

However, the development of Cas12a-based programmable biosensing systems using amplification techniques such as conventional RPA or PCR could be seriously hindered by the PAM requirement. The presence of a PAM is mandatory for the Cas12a activation by a dsDNA target since Cas12a originally binds to dsDNA at a PAM site. This binding initiates the subsequent steps of molecular recognition, such as local destabilization of the DNA duplex, followed by the formation of an R-loop with RNA:DNA heteroduplex via gRNA spacer invasion [33]. A perfect or near-perfect spacer–protospacer pairing results in a Cas12a activation. For recognition to occur, a PAM site has to be located right next to the 5′-end of the sequence complementary to the protospacer sequence in dsDNA. In the case of Cas12a nuclease, the consensus PAM sequence is “TTTN” [34]. Although, for a dsDNA amplicon of more than a hundred bp in length, the probability of finding at least a single stretch of three thymidines approaches unity, the probability of finding it in a desirable location in such an amplicon would be about 1%, thus severely constraining the choice of amplicon sections for gRNA targeting. In contrast, for ssDNA, the formation of “spacer–protospacer” duplex can occur directly and does not require steps of the initial Cas12a binding, dsDNA destabilization, and R-looping. That makes the PAM presence unnecessary. Thus, switching from dsDNA to ssDNA as a target appears to be a universal solution to the PAM problem. The successful implementation of such a solution based on the use of asymmetric amplification is demonstrated in a number of recent publications [10,11,12,13,14,15].

The conventional PCR is commonly assumed to produce dsDNA amplicons. By making the amplification asymmetric, ssDNA amplicons can be generated alongside the dsDNA amplicons and can consequently induce the Cas12a activation even in the absence of a PAM. aPCR has been known for decades and has broadly been used to produce ssDNA for various applications [35,36]. The yield of ssDNA in aPCR depends in a complex manner on several parameters [37,38]. Such parameters include primers’ *T*_m_ values and *T*_m_ difference (Δ*T*_m_), the annealing temperature, primer molar ratio and primer absolute concentrations [35,37,38]. In fact, in many presently existing PCR tests, *T*_m_ values of primers are not exactly the same and differ to a greater or lesser extent. However, to make a PCR with an equal primer molar ratio truly asymmetric, the primers’ Δ*T*_m_ has to exceed 15 °C [35]. In the particular case of the Y1 and Y2 primers, the melting temperatures of the primers are, respectively, 66 °C and 59 °C, as calculated with the online Oligonucleotide Properties Calculator (https://oligocalc.eu/, accessed on 23 March 2026). As a result, no appreciable amount of ssDNA has been produced at the Y1/Y2 molar ratio of 1 (Figure 2b). By progressively shifting the equilibrium toward the synthesis of the target DNA with the increase in the Y1/Y2 molar ratio, the Cas12a nuclease was effectively activated by aPCR products. The activation reached a maximum in the Y1/Y2 molar ratio range of 4 to 8 but rapidly declined at ratios above 10. That likely reflects the sharp increase in *C*_q_ values at Y1/Y2 molar ratios above 10 (Figure 2a and Figure S6). As a result, peak dsDNA amplicon production is delayed and more cycles are required to generate single-stranded amplicons in sufficient amounts, using dsDNA amplicons as a template. For a fixed number of aPCR cycles, this should lead to a lower amount of ssDNA amplicons. As expected, when the synthesis of the non-target strand has been favored by increasing the Y2/Y1 molar ratio, no Cas12a activation has been observed.

The post-PCR heat treatment of the mostly double-stranded amplicons produced in PCR with equimolar primer concentrations resulted in an effective Cas12a activation (Figure 2b). Such technically simple treatment has recently been suggested to couple the downstream Cas12a analysis to RPA or conventional end-point PCR [39] and may represent a valuable alternative to aPCR to circumvent the PAM requirement. However, this approach relies on incomplete reannealing of amplicon strands. This incompleteness may be temporal and vary substantially, depending on the amplicon’s length and sequence. Indeed, the reannealing of amplicon strands is a particular case of DNA hybridization for which effects of length and sequence are well known (reviewed in [40]). DNA strands can fold upon themselves to form various intramolecular structures (hairpins, stems-loops, pseudoknots, etc.), thus slowing the kinetics of reannealing and leaving some sections of DNA strands in a single-stranded state for some time [40]. Clearly, the shorter the amplicons, the quicker and more efficiently they reanneal. In contrast, the use of aPCR appears as a more general approach to designing PAM-independent Cas12a-based biosensing systems. By simply varying the primer molar ratio, the substantial and stable yield of ssDNA can be obtained.

Ideally, parameters governing the ssDNA production in aPCR have to be carefully evaluated to maximize its yield. However, in practical terms, for coupling the Cas12a-based analysis to aPCR, the yield does not need to be maximal but rather sufficient for an efficient Cas12a activation. That makes a thorough optimization of aPCR unnecessary, thus simplifying the design of aPCR/Cas12a detection systems. In our case, concentrations of 400 nM and 100 nM for Y1 and Y2 primers, respectively, were accepted as suitable for production of the target DNA strand in sufficient amounts. The other PCR parameters, except for a cycle duration, have been left unchanged.

As was mentioned in the Introduction, Cas12a nucleases are not thermophilic and would not tolerate the high temperatures used in PCR. That assumes that an aPCR/Cas12a biosensing system cannot be designed as a one-pot assay and Cas12a analysis has to be done post-PCR in a separate test tube. The most important issue resolved with one-pot assays is that there is no need for transferring amplification products from one reaction tube to another. That prevents aerosol contamination of the work area with amplicons. One of the ways to solve the problem of cross-contamination by PCR products is to modify amplicons by inserting deoxyuridines into DNA during PCR [32]. The consequent introduction of uracil-DNA glycosylase (UDG) treatment into a PCR protocol as a preliminary step prior to amplification (dUTP-UDG-PCR) results in degradation of cross-contaminating modified amplicons. Previously, the ability of Cas12a nuclease to recognize uracil-containing amplification products has been demonstrated for LAMP amplicons [41]. Yet, in LAMP, the yield of amplification products is 10-fold higher than that in conventional PCR. In aPCR, the yield of ssDNA amplicons may be even lower. Furthermore, the activation has been demonstrated for Cas12a nuclease from a *Lachnospiraceae bacterium*. Since Cas12a orthologs can be known to exhibit variability in specific characteristics of DNA recognition [33], we successfully tested the ability of Cas12a nuclease from *Francisella tularensis*, used in this study, to be activated by uracil-containing ssDNA amplicons generated in aPCR. Thus, the probability of false-positive results due to cross-contamination can be effectively decreased for the aPCR/Cas12a biosensing system if home-made or commercial dUTP- and UDG-containing PCR mixtures are employed for aPCR.

The designed aPCR/Cas12a biosensing system detects *P. polaris* represented by the strain B-3420 (Table S1) with high sensitivity. The detection sensitivity was determined by that of aPCR and a physical limit of detection as low as one copy of bacterial genome per reaction can be reached. However, at such a load, the detection was irreproducible. Since the analytical LOD is defined as a minimal amount of reliably detected analyte [42], it has been set at 10 copies of *P. polaris* genome per PCR. For that load, the detection was highly reproducible—10 genome copies were constantly detected in 32 consecutive aPCR/Cas12a-based tests. It should be noted that this LOD is 20 and 2000 times lower than the LODs reported to date for *P. polaris* detection: 200 and 20,000 genome copies per PCR with the endpoint and real-time PCR, respectively [27]. In addition, the endpoint PCR with a LOD of 200 copies of *P. polaris* genome required the electrophoretic analysis of PCR products [27], which is time-consuming and hardly compatible with on-site NAT. The overall time of the aPCR/Cas12a-based testing with visual or instrumental detection was about 2 h, which is compatible with the duration of routine PCR analysis.

The analytical performance of the designed aPCR/Cas12a detection system was determined using a single strain of *P. polaris*. To validate the analytical specificity for *P. polaris* as a species, a substantial number of strains of this pathogen has to be successfully tested. Nonetheless, it is worth noting that, for four strains of *P. polaris* (strains NIBIO1392, NIBIO1006, BP9026, and QK413-1), the complete genome sequences are available from the NCBI database (https://www.ncbi.nlm.nih.gov, accessed on 15 June 2026; the accession numbers NZ_CP017482.1, CP017481.1, CP189681.1, and CP077421.1, respectively). The sequences of sections targeted by gRNAs in our work are identical among these strains. These sequences are also identical to the sequence targeted with gRNA3 in amplicons produced by aPCR with the Y1–Y2 primer pair, using genomic DNA of *P. polaris* strain B-3420 as a template (Table S3). Thus, the Cas12a/gRNA3 complex would recognize the Y1–Y2 ssDNA amplicons from at least five strains with similar efficiency. The selectivity of the Y1–Y2 primer pair, per se, was demonstrated in numerous publications (the original paper of Darrasse et al. [18] and, e.g., [43,44,45,46], to mention a few). Nonetheless, experimental testing of more *P. polaris* strains is still required to position the designed aPCR/Cas12a biosensing system as a practical, ready-to-use assay for *P. polaris* detection.

The Cas12a-based analysis of amplicons can, in general, either provide or enhance detection selectivity. For the genus-specific Y1–Y2 primer pair, the Cas12a analysis of ssDNA amplicons allowed the *P. polaris* strain B-3420 to be differentiated from strains of other *Pectobacterium* species. Since for ssDNA, the sequence of the gRNA spacer is no longer predefined by a PAM site location, the particular section of amplicons has been selected for gRNA targeting. Compared to amplicons from *P. polaris*, there are multiple nucleotide substitutions in this section for other *Pectobacterium* species. By priming the gRNA spacer to different positions within this section, the detection selectivity was fine-tuned so as to completely discriminate all other *Pectobacterium* species by the rate of MR cleavage. That can be thought to be especially important for visual detection. Indeed, while instrumental detection allows one to reliably differentiate *P. polaris* from other *Pectobacterium* species by a shape of MR cleavage kinetic curves or mean *V*_0_ values (Figure 5 and Figure 6), it may not be easy to distinguish shades of color in the case of visual detection. With gRNA3, which provided the best selectivity, the differences in color were undoubtable (Figure 8 and Figure 9).

Presently, there are no rationales for a choice of a particular sequence for priming of the gRNA spacer. From general considerations, the more nucleotide substitutions in the spacer-primed sequence, the better non-target species would be discriminated since mismatches in the spacer/protospacer duplex are known to affect functional activities of Cas12a nucleases [47,48]. However, *trans*- and *cis*-activities can be altered in different ways by a mismatch. For example, in the case of Cas12a nuclease from *Francisella tularensis*, a mismatch in the seed region (first eight 3′-end nucleotides of gRNA) has been reported to increase the Cas12a *trans*-cleavage activity while decreasing on-target (*cis-*) activity [49]. In general, the Cas12a *trans*-cleavage activity is known to depend in a complex and, in many instances, unpredictable manner on the number of mismatches, their positions, spacer length, and even spacer sequence, per se [13,50,51,52]. Currently, the choice of spacer-priming site has to be done empirically on a case-by-case basis.

As a discussion point, the aPCR/Cas12a biosensing system adapted for on-site NAT in the present study has the potential to be developed into a field-deployable system. Presently, there are commercially available autonomous (powered by rechargeable batteries) handheld thermocyclers [53]. However, the most low-cost and technically simple solutions rely on the use of convective PCR [54]. In convective PCR, programmable thermal cycles are replaced with thermal cycling relying on buoyancy-driven fluid circulation (convection). Such circulation requires the reaction mixture viscosity to be fine-tuned. Furthermore, in convective PCR, fast DNA polymerases are usually needed to make amplification efficient [54]. The Cas12a-based analysis of aPCR products should not be affected by these modifications of aPCR mixtures. At the same time, to conduct asymmetric dUTP-UDG-PCR as convective aPCR, a careful re-evaluation and adjustment of aPCR conditions would be required. This is believed to be more of a subject of a dedicated study.

## 5. Conclusions

The present study demonstrates that aPCR/Cas12a biosensing can be adapted to the requirements of on-site nucleic acid testing without compromising its sensitivity and selectivity. aPCR runs can be conducted on a low-cost, portable thermocycler in an endpoint detection mode, producing uracil-containing single-stranded amplicons in amounts appropriate for efficient activation of Cas12a nuclease. Uracil incorporation followed by uracil-DNA glycosylase treatment would prevent false positives caused by potential carryover amplicon contamination. This may be especially important for on-site testing, where no highly trained technical personnel are assumed to be involved and the work area cannot necessarily be zoned. The uracil-containing amplicons produced in aPCR activate the Cas12a nuclease to a level sufficient for a visual readout in a practical timeframe. Even with long amplicons such as those for aPCR with the Y1–Y2 primer pair against *Pectobacterium* spp. (435 nucleotides), the overall testing time was about 2 h, which is compatible with the duration of routine PCR analysis.

Though *Pectobacterium* species, and, in particular, *P. polaris*, were employed as a convenient model, the study’s findings may provide a foundation for developing next-generation assays for on-site detection of these pathogens. The designed aPCR/Cas12a biosensing system selectively detects *P. polaris* with a LOD of 10 genome copies per reaction, exceeding the LOD of other methods by at least 20-fold. The absence of a PAM requirement allowed for fine-tuning the selectivity of the Cas12a assay against *P. polaris* by properly positioning the gRNA spacer on the ssDNA amplicons. Still, more strains of *P. polaris* must be successfully tested to position the designed aPCR/Cas12a detection system as a practical ready-to-use assay.

In a broader context, it is believed that a considerable number of current PCR tests can be easily adapted to a format of aPCR/Cas12a programmable biosensing by simply varying the primer molar ratio to convert PCR into aPCR and by testing PCR products with Cas12a/gRNA complexes to find the acceptable rate of MR cleavage for visual detection. This may pave the way for modifying current laboratory-based PCR tests into on-site, aPCR/Cas12a-based tests when combined with low-cost, portable thermocyclers.

## Figures and Tables

**Table 1:** The sequences of gRNAs. The spacer sequences are shown in italics.

Name	Sequence (5′→3′)
gRNA1	GGGAAUUUCUACUGUUGUAGAU*CAUCAGCAUAUUGUCUGUUG*
gRNA2	GGGAAUUUCUACUGUUGUAGAU*GCGAGAUCCACUUUGGUGUC*
gRNA3	GGGAAUUUCUACUGUUGUAGAU*GAUCCACUUUGGUGUCUUUA*
gRNA4	GGGAAUUUCUACUGUUGUAGAU*CCACUUUGGUGUCUUUACCC*

**Table 2:** Asymmetric PCR with different durations of cycle steps. The Y1/Y2 molar ratio = 2; 200 copies of *P. polaris* genome per PCR.

PCR Regime (Denaturation–Annealing–Extension)	Mean Cq Value	The Duration of 60 Cycle Run, h
“60–60–90”	24	4.1
“45–45–60”	25	3.0
“30–30–45”	27	2.3
“15–15–30”	29	1.5

## Data Availability

Data is contained within the article and Supplementary Materials.

## Supplementary Materials

The following supporting information can be downloaded at: https://www.mdpi.com/article/10.3390/bios16090512/s1.

## Abbreviations

The following abbreviations are used in this manuscript:

aPCR	asymmetric polymerase chain reaction
RPA	recombinase polymerase amplification
LAMP	loop-mediated isothermal amplification
ssDNA	single-stranded DNA
dsDNA	double-stranded DNA
gRNA	guide RNA
PAM	protospacer adjacent motif
LOD	limit of detection
PCR NTC	PCR no-template control
dUTP	2′-deoxyuridine 5′-triphosphate
dTTP	2′-deoxythymidine-5′-triphosphate
