# Supplementary material for: Combining Asymmetric PCR with PAM-Independent Cas12a Analysis of Single-Stranded Amplicons for On-Site Nucleic Acid Testing
Source: Biosensors (Basel). 2026 Sep 11;16(9):512. doi: 10.3390/bios16090512 (PMC13604837; doi:10.3390/bios16090512)
Supplement: Supplementary file 1 [file biosensors-16-00512-s001.zip › Supplementary Materials_Figures S1-S14_Tables S1, S2, S4.pdf]

Supplementary Materials

for

Combining asymmetric PCR with PAM-independent Cas12a analysis of single-stranded amplicons for on-site nucleic acid testing

Konstantin G. Ptitsyn [1], Olga S. Timoshenko [1], Svetlana A. Khmeleva [1], Leonid K. Kurbatov [1], Adelina A. Stepanova [1], Evgenia M. Nikiforova [1], Elena V. Suprun [2], Sergey P. Radko [1,*] and Andrey V. Lisitsa [1]

[1] V.N. Orekhovich Institute of Biomedical Chemistry, 10 Pogodinskaya St., 119121 Moscow, Russia

[2] The Chemistry Faculty of the M.V. Lomonosov Moscow State University, 1/3 Lenin Hills, 119991 Moscow, Russia

* Correspondence: radkos@yandex.ru

Table S1. The list of strains used in the study. Strains are from the All-Russian Collection of Microorganisms (VKM), the German Collection of Microorganisms and Cell Cultures (DSMZ), and the Collection of the All-Russian Scientific Research Institute of Phytopathology (VNIIF).

**Species**	**Host**	**Origin**	**Collection**	**Strain Number**
Pectobacterium versatile	potato	Russia	VKM	B-3416
Pectobacterium aquaticum	potato	Russia	VKM	B-3417
Pectobacterium polaris	potato	Russia	VKM	B-3420
Pectobacterium parmentieri	potato	Russia	VKM	B-3423
Pectobacterium carotovorum	potato	Denmark	VKM	B-1247
Pectobacterium brasiliense	potato	Russia	VKM	B-3424
Pectobacterium brasiliense	potato	Russia	VKM	B-3425
Pectobacterium odoriferum	potato	Russia	VNIIF	1557
Clavibacter sepedonicus	potato	USA	VKM	Ac-1405
Clavibacter michiganensis	tomato	USA	VKM	Ac-1403
Clavibacter michiganensis	tomato	Zambia	VKM	Ac-1144
Clavibacter phaseoli	common beans	Spain	VKM	Ac-2641
Clavibacter insidiosus	alfalfa	USA	VKM	Ac-1402T
Clavibacter nebraskensis	maize	USA	VKM	Ac-1404T
Clavibacter tesselarius	wheat	USA	VKM	Ac-1406T
Dickeya chrysonthemi	Chrysanthemum morifolium	USA	DSMZ	DSM 4610
Dickeya solani	potato	Russia	VNIIF	1C3
Agrobacterium tumefaciens	undefined	USA	VKM	B-1573
Escherichia coli	clinical isolate	USA	VKM	B-3034

Table S2. Sequences of oligonucleotides used in the study: PCR primers (Y1 and Y2), DNA templates for enzymatic synthesis of gRNAs, the oligonucleotide for construction of T7 promotor (T7P), and molecular reporter (MR; FAM – 6-carboxyfluorescein, BHQ1 – black hole quencher 1).

Name	Sequence (5′→ 3′)
Y1	TTACCGGACGCCGAGCTGTGGCGT
Y2	CAGGAAGATGTCGTTATCGCGAGT
gRNA1_template	CAACAGACAATATGCTGATGATCTACAACAGTAGAAATTCCC CTATAGTGAGTCGTATTA
gRNA2_template	GACACCAAAGTGGATCTCGCATCTACAACAGTAGAAATTCCC CTATAGTGAGTCGTATTA
gRNA3_template	TAAAGACACCAAAGTGGATCATCTACAACAGTAGAAATTCCC CTATAGTGAGTCGTATTA
gRNA4_template	GGGTAAAGACACCAAAGTGGATCTACAACAGTAGAAATTCCC CTATAGTGAGTCGTATTA
T7P	TAATACGACTCACTATAGGG
MR	FAM-TTATTATTAT-BHQ1

Table S3. The sequences of the amplified region in genomes of *Pectobacterium* species, targeted with primers Y1 and Y2.

Refer to the accompanying excel file.

Table S4. Detection of *P. polaris* in artificially contaminated potato tuber samples by real-time PCR and with the aPCR/Cas12a assay. The values of C_q are means of duplicate measurements. The results of the aPCR/Cas12a assay are given as positive (+) or negative (–), based on the MR cleavage kinetics. All tests are conducted with a load of 200 ng of total DNA composed of potato DNA and a trace amount of bacterial DNA. Real-time PCR is run for 50 cycles, using the commercial kit for *Pectobacterium* spp. detection, as recommended by the kit manufacturer. The number of *P. polaris* genome copies was determined with the standard curve provided by the manufacturer of the kit. ND – not defined. The optimized aPCR is run for 60 cycles.

**Potato sample type**	C_q	**genome copies per PCR reaction**	**aPCR/Cas12a detection**
contaminated with *P. polaris*	25.5	147 000	+
	28.7	18900	+
	32.8	1480	+
	35.7	215	+
	39.9	15	+
not contaminated with *P. polaris*	ND	0	–
	ND	0	–
	ND	0	–

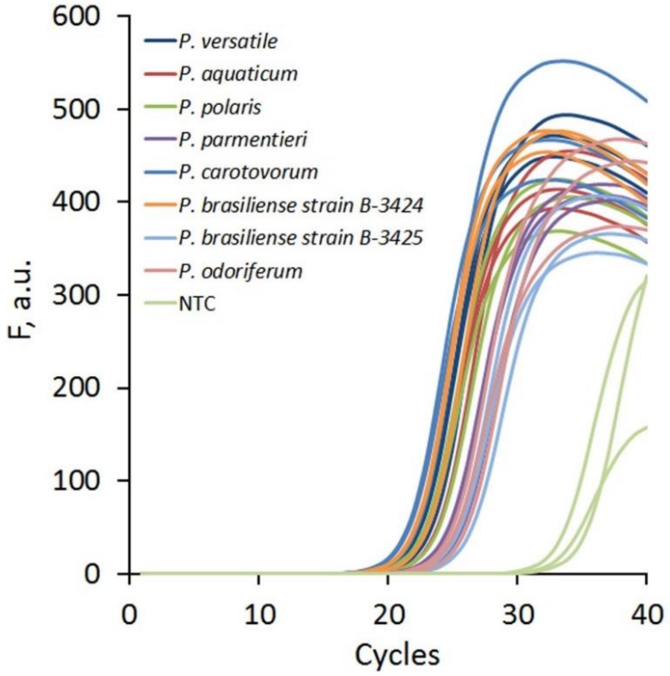

Figure S1. PCR analysis of *Pectobacterium* species. PCR regime – "60-60-90"; the Y1/Y2 molar ratio of 1; the load of bacterial DNA – 1 pg per PCR reaction. PCR runs are conducted in triplicates. F – fluorescence in arbitrary units (a.u.). *Pectobacterium* species are indicated by the curve color. NTC – no template control.

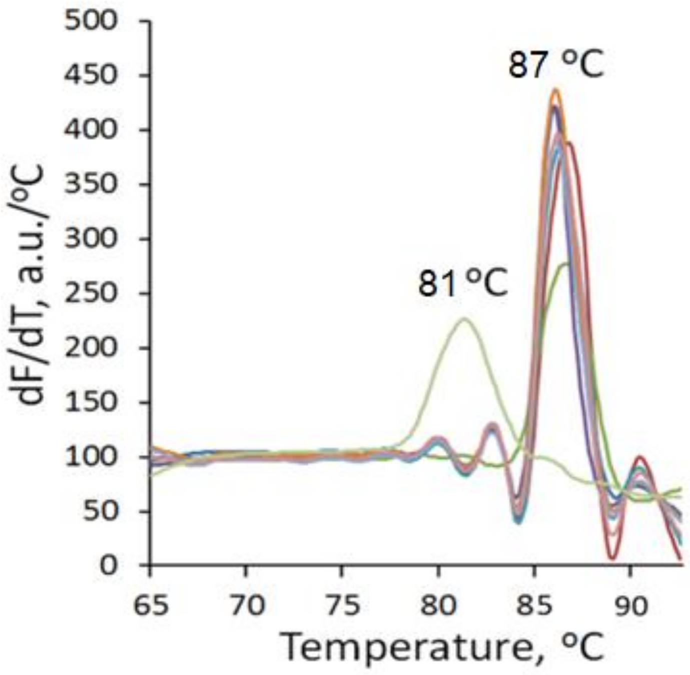

Figure S2. The melting curve analysis of PCR products. dF/dT – the first derivative of fluorescence F (in arbitrary units, a.u.) as a function of temperature. The representative melting curves for amplicons generated in conventional PCR in Figure S1. The color designation as in Figure S1. The melting temperatures are indicated in the figure.

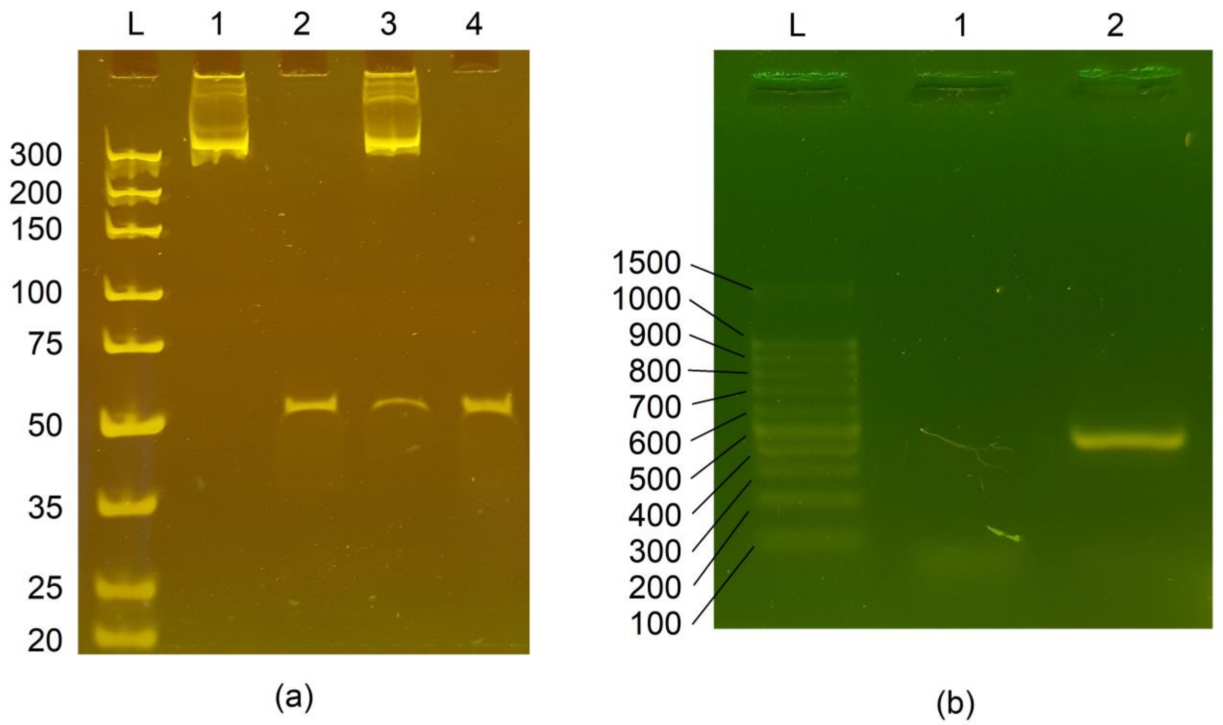

Figure S3. Electrophoretic analysis of PCR and aPCR products. Gels are stained with SYBR green I fluorescent dye. (a) Polyacrylamide gel (8%) electrophoresis in Tris-borate buffer. L – DNA size standards (size in base pairs is indicated on the left); 1 and 2 – the 40-cycle conventional PCR (Y1/Y2 molar ratio = 1, the amplification regime “60-60-90”) with 10 copies of *P. polaris* genome and no template control, respectively; 3 and 4 – the 60-cycle optimized aPCR (Y1/Y2 molar ratio = 4, amplification regime “15-15-30”) with 10 copies of *P. polaris* genome and no template control, respectively. (b) Agarose gel (2%) electrophoresis in Tris-acetate-EDTA buffer. L – DNA size standards (size in base pairs is indicated on the left); 1 and 2 – the 60-cycle optimized aPCR (Y1/Y2 molar ratio = 4, amplification regime “15-15-30”) with 10 copies of *P. polaris* genome and no template control, respectively.

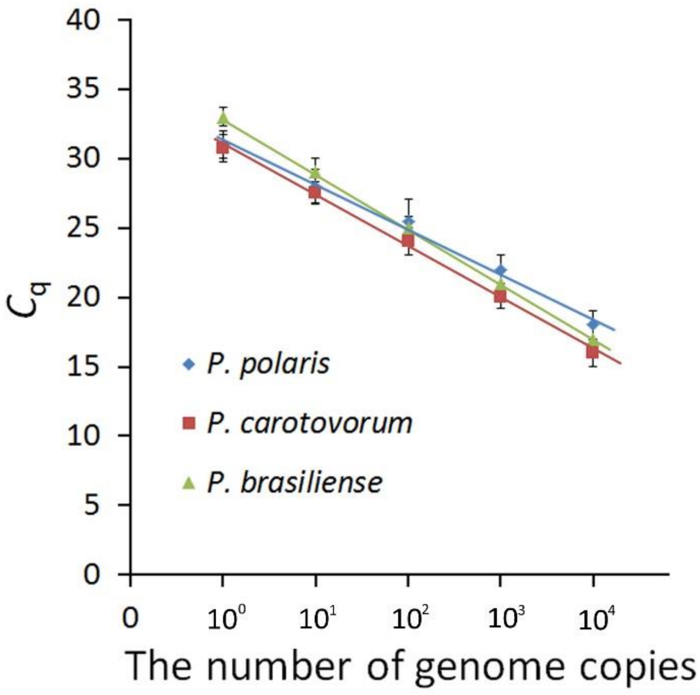

Figure S4. Dependencies of quantification cycle (C_q) on the number of genome copies per PCR reaction for *P. polaris*, *P. carotovorum*, and *P. brasilience* strain B-3424. PCR with the Y1/Y2 molar ratio of 1, amplification regime "60-60-90". The C_q mean value and the corresponding standard deviations are shown.

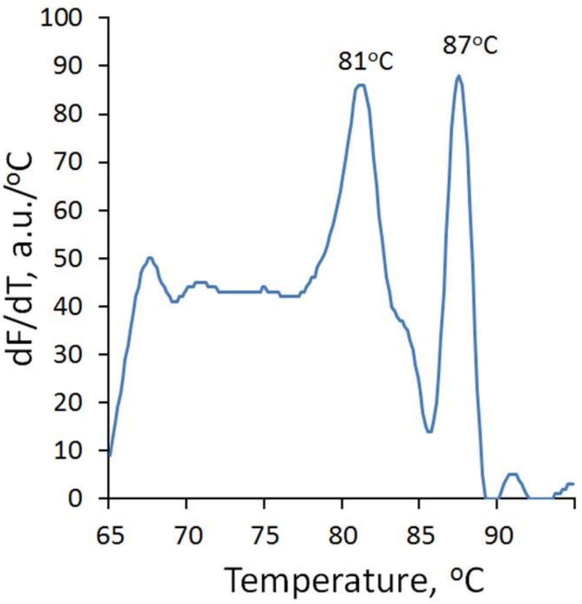

Figure S5. The representative melting curve. dF/dT – the first derivative of fluorescence F (in arbitrary units, a.u.) as a function of temperature. The 40-cycle PCR: Y1/Y2 molar ratio = 1, amplification regime "60-60-90". DNA load – 1 copy of *P. polaris* genome. The melting temperatures are indicated in the figure.

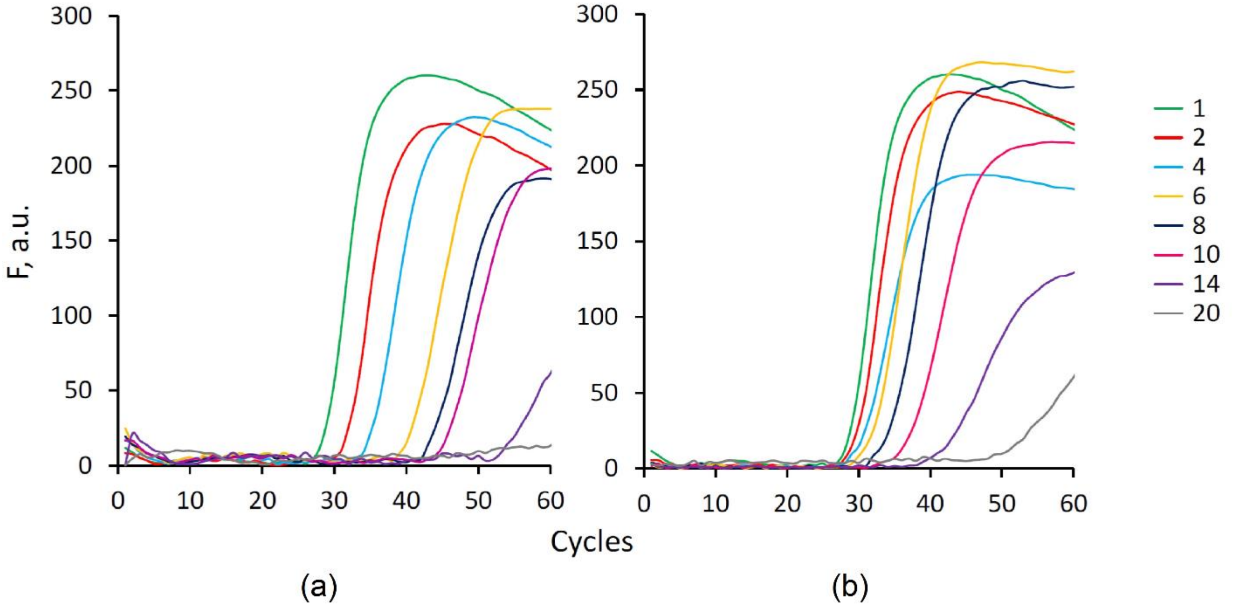

Figure S6. The impact of primer molar ratio on aPCR performance. Representative amplification curves. The molar ratios are indicated by color as shown on the right. (a) Y1/Y2 molar ratios. (b) Y2/Y1 molar ratios. The other conditions as in Figure 2a.

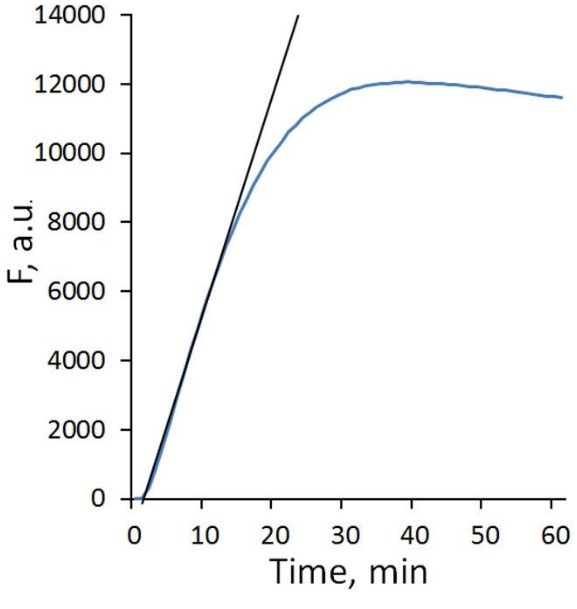

Figure S7. Determination of the initial rate of MR cleavage, V_0, as a slope of the near-linear increase of fluorescence at the beginning of a kinetic curve. F – fluorescence in arbitrary units (a.u.). The near-linear increase of fluorescent and the slope are indicated by a black straight line. The representative example.

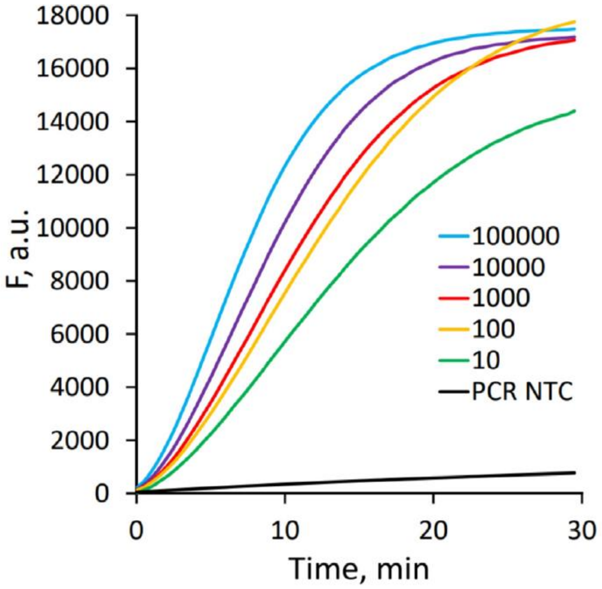

Figure S8. Representative fluorescence kinetic curves for MR cleavage by the Cas12a nuclease activated with aPCR products. 1 µL of the completed aPCR reaction per 20 µL of the Cas12a reaction mixture, 37 °C. The 60-cycle aPCR runs conducted with different loads of *P. polaris* DNA. The loads are indicated in the figure as the number of genome copies per aPCR reaction. F – fluorescence in arbitrary units (a.u.).

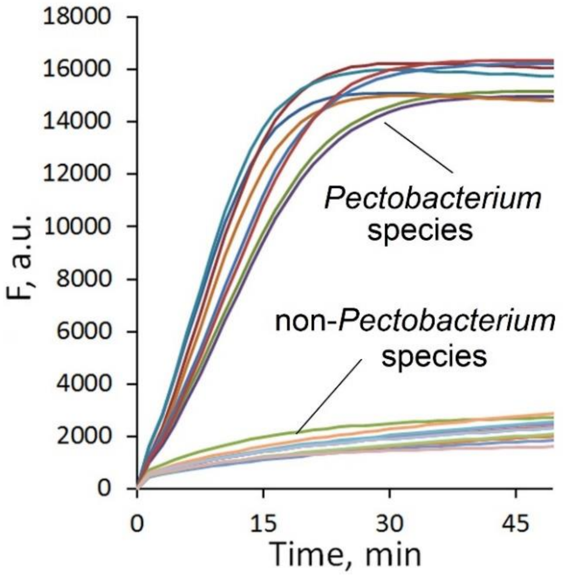

Figure S9. The representative curves of MR cleavage by activated Cas12a nuclease. *Pectobacterium* and non-*Pectobacterium* species from Table S1. F – fluorescence in arbitrary units (a.u.). Cas12a nuclease in a complex with gRNA1 was activated by ssDNA amplicons generated with the optimized aPCR (PCR regime – "15-15-60", Y1/Y2 molar ratio = 4). 1 µL of the completed aPCR reaction per 20 µL of the Cas12a reaction mixture, 37 °C. aPCR was conducted with 0.05 pg of bacterial genomes per PCR reaction for *Pectobacterium* species and with 0.5 ng of bacterial DNA per PCR reaction for non-*Pectobacterium* species.

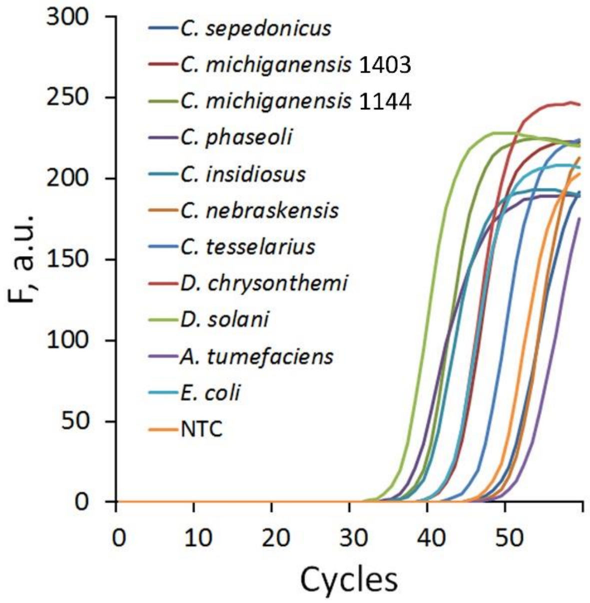

Figure S10. PCR analysis of non-*Pectobacterium* species. Representative amplification curves. F – fluorescence in arbitrary units (a.u.). PCR regime – “15-15-60”; Y1/Y2 molar ratio = 4; the load of bacterial DNA – 0.5 ng per PCR reaction. The non-*Pectobacterium* species tested are indicated in the figure by the curve color. NTC – no template control.

**Species**	*Nucleotide position in amplicon's sequences (5' to 3')*																										
	78	79	80	81	82	83	84	85	86	87	88	89	90	91	92	93	94	95	96	97	98	99	100	101	102	103	104
P. polaris	G	G	G	T	A	A	A	G	A	C	A	C	C	A	A	A	G	T	G	G	A	T	C	T	C	G	C
P. versitale	G	G	G	T	A	A	A	G	A	C	G	T	T	A	A	A	G	T	G	G	A	T	C	T	C	G	C
P. aquaticum	G	G	G	T	A	A	A	G	A	G	G	T	T	A	A	A	G	T	G	G	A	C	C	T	C	G	C
P. parmentieri	G	G	G	C	A	A	G	G	A	C	G	T	C	A	A	A	G	T	A	G	A	T	C	T	C	G	C
P. carotovorum	G	G	G	T	A	A	A	G	A	C	G	T	T	A	A	A	G	T	G	G	A	T	C	T	C	G	C
P. brasiliense	G	G	G	T	A	A	A	G	A	C	G	T	T	A	A	A	G	T	G	G	A	T	C	T	C	G	C
P. odoriferum	G	G	G	T	A	A	A	G	A	C	G	T	T	A	A	A	G	T	G	G	A	T	C	T	C	G	C
	Sequences of gRNA spacers (3' to 5')																										
gRNA2								C	U	G	U	G	G	U	U	U	C	A	C	C	U	A	G	A	G	C	G
gRNA3				A	U	U	U	C	U	G	U	G	G	U	U	U	C	A	C	C	U	A	G				
gRNA4	C	C	C	A	U	U	U	C	U	G	U	G	G	U	U	U	C	A	C	C							

Figure S11. The 27-nucleotide long segment of amplicons targeted by gRNA2, gRNA3, and gRNA4. Nucleotide mismatches are indicated by red letters.

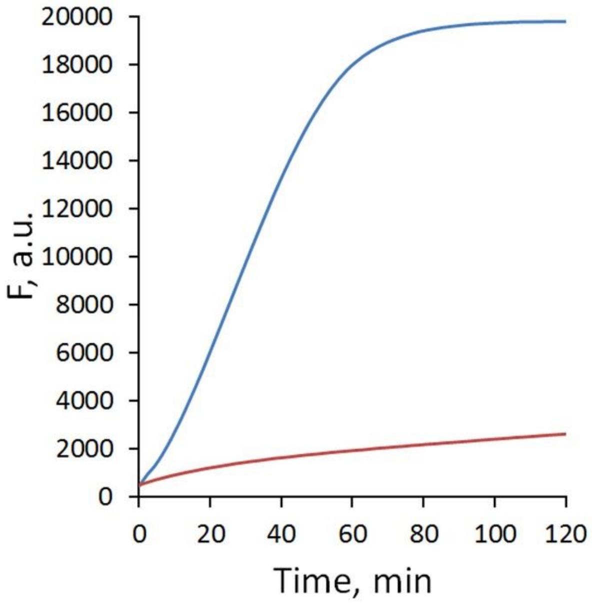

Figure S12. The representative curves of MR cleavage by activated Cas12a nuclease. Blue colored curve – The Cas12a nuclease in a complex with gRNA3 activated by uracil-containing ssDNA amplicons. Amplicons were generated by the optimized aPCR with 50% substitution of dUTP for dTTP in the PCR reaction mixture. Red colored curve – PCR NTC. PCR regime – "15-15-60", Y1/Y2 molar ratio = 4; 10 copies of *P. polaris* genome per PCR reaction. 1 μL of the completed aPCR reaction per 20 μL of the Cas12a reaction mixture, 37 °C. F – fluorescence in arbitrary units (a.u.).

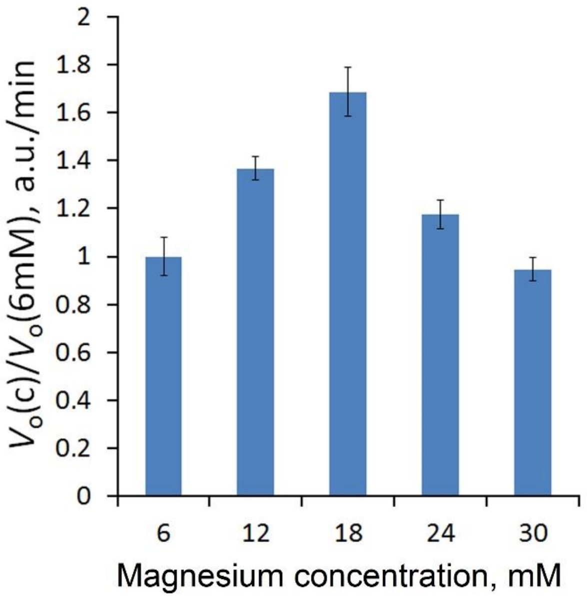

Figure S13. The impact of magnesium concentration in the Cas12a reaction mixture on the relative initial rate of MR cleavage by the activated Cas12a nuclease. $V_0(c)$ and $V_0(6\text{ mM})$ – the initial rates at magnesium concentrations of c and 6 mM, respectively. The other conditions as in Figure S10.

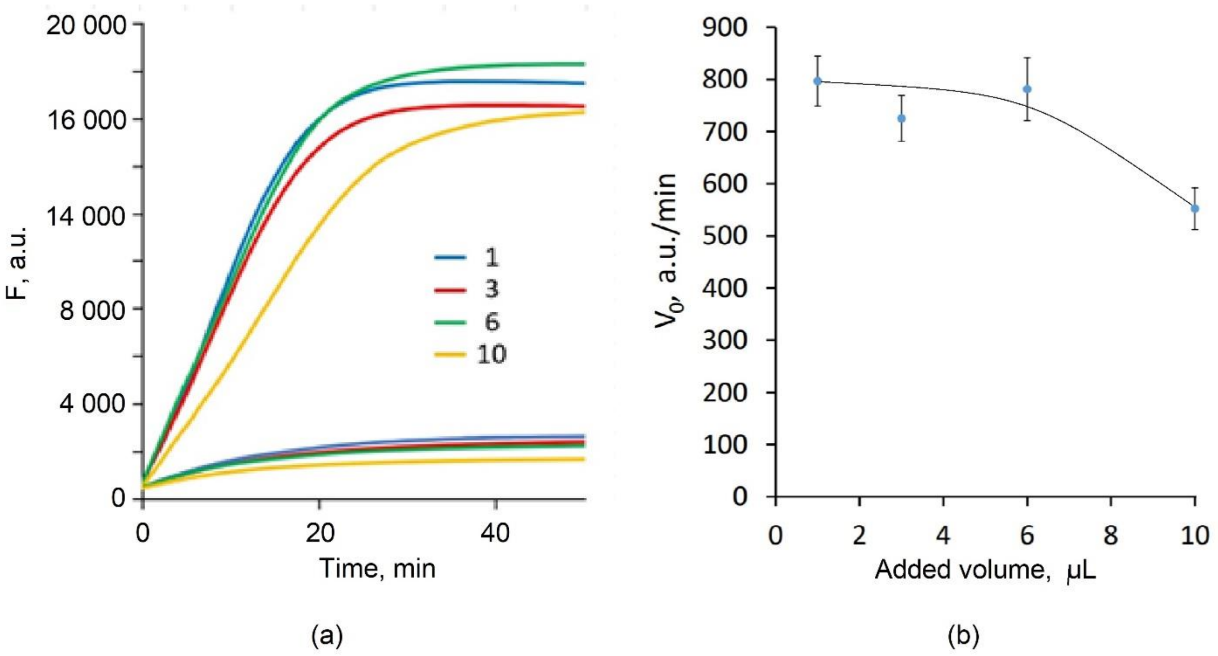

Figure S14. The effect of the added volume of completed aPCR reaction on MR cleavage by Cas12a nuclease. (a) Representative kinetic curves. The volumes (μL) of aPCR reaction, added to the Cas12a reaction mixture, are shown in the figure and indicted by the curve color. (b) The initial cleavage rate, V_0, as a function of the added volume. The mean values and the corresponding standard deviations for three measurements are shown. The other conditions as in Figure S11, exept for the magnesium concetration which was 18 mM.
